# Genome‐Wide Population Structure of Lake Whitefish (*Coregonus clupeaformis*) in a Subarctic Great Lake

**DOI:** 10.1111/eva.70268

**Published:** 2026-05-28

**Authors:** Philippe Hénault, Raphaël Bouchard, David A. Boguski, Brendan K. Malley, Eric Normandeau, Charles Babin, Xavier Dallaire, Louis Bernatchez, Xinhua Zhu, Jean‐Sébastien Moore

**Affiliations:** ^1^ Institut de Biologie Intégrative et Des Systèmes (IBIS) Université Laval Québec Québec Canada; ^2^ Département de Biologie Université Laval Québec Québec Canada; ^3^ Fisheries and Oceans Canada, Arctic Fisheries and Mammal Science Division Freshwater Institute Winnipeg Manitoba Canada; ^4^ Plateforme de Bio‐Informatique de L'IBIS (Institut de Biologie Intégrative et des Systèmes) Université Laval Québec Québec Canada

**Keywords:** conservation genomics, genetic structure, low‐coverage whole‐genome resequencing, northern Great Lakes, salmonid, structural variation

## Abstract

Advances in genomics have facilitated the delineation of fisheries management units, which can be challenging in systems such as large lakes, in which high gene flow tends to limit genetic structure. In Great Slave Lake, Lake Whitefish populations have supported an important commercial fishery since the mid‐1940s. The genetic structure of Lake Whitefish, however, has never been assessed, preventing the implementation of population‐specific monitoring. Using low‐coverage whole‐genome resequencing of 305 samples from 10 sampling locations, we identified eight genetically differentiated populations of Lake Whitefish in Great Slave Lake and its main tributary, the Slave River. In the lake, we observed elevated levels of genetic differentiation among putative spawning locations in environmentally heterogeneous sections of the Main Basin despite small geographic distances among sites. In contrast, we observed weak genetic structure between populations in the comparatively homogeneous East Arm despite large geographic distances. Our observations suggest that mechanisms such as spawning site fidelity, adfluvial migratory behaviour, or local adaptation might shape population structure in this system. Using genome‐wide scans, we found multiple genomic regions of elevated differentiation, with some displaying patterns coherent with chromosomal inversions. These results highlight the potential role of chromosomal rearrangements in maintaining local adaptation in the face of gene flow in environmentally heterogeneous lakes. Overall, our study provides novel insights into the genetic structure of fish populations in vast, recently deglaciated lakes. Furthermore, our results highlight the power of genomic data for population delineation in systems with high gene flow. Lastly, our precise assessment of genetic structure will provide a baseline for the genetic monitoring of culturally and socio‐economically important Lake Whitefish commercial fisheries in this subarctic Great Lake.

## 1 Introduction

The conservation of intraspecific diversity is of crucial importance to the long‐term persistence of species and their contribution to ecosystems and to people's livelihoods and well‐being (Des Roches et al. 2021; Luck et al. 2003). Advancements in population genomics have improved the resolution of population structure analyses and the detection of genomic regions under selection, allowing for a deeper understanding of how genetic variation is maintained and distributed in populations (Allendorf et al. 2010). This has benefited wildlife conservation as genomics provides crucial information on population connectivity (e.g., Bouchard et al. 2025) and local adaptation (e.g., Xuereb et al. 2022) that can be used to define conservation and management units (Funk et al. 2012; Hohenlohe et al. 2021; Forester et al. 2022; Lehnert et al. 2023).

The classification of harvested fish into management units is especially important to help prevent the negative impact of commercial fishing on genetic diversity and natural population subdivisions (Allendorf et al. 2008; Laikre and Ryman 1996; Delgado et al. 2025). In fisheries, the genetic delineation of management units (i.e., stocks) has long been challenging in systems with high gene flow (e.g., marine systems; Waples 1998). Recently, genome‐wide resolution has enabled the delineation of weakly structured populations, promoting precise monitoring of species using genetic stock identification and mixed‐stock analysis (Bernatchez et al. 2017; Andersson et al. 2024). For instance, genome‐wide data can be used to design SNP panels that target highly differentiated regions of the genome that can accurately discriminate weakly structured populations (Beemelmanns et al. 2025; Bootsma et al. 2020; McKinney et al. 2020) and can reveal the presence of large‐effect loci that can be integrated in conservation measures of evolutionarily significant units (Schneller et al. 2025; Waples et al. 2020).

In North America, many large lakes support important mixed‐stock commercial fisheries (Brenden et al. 2012), which would benefit from genomic monitoring initiatives (Ebener et al. 2021) to prevent the overharvesting of populations with lower productivity (e.g., Molton et al. 2013) and enhance ecosystem and fishery stability through the portfolio effect (DuFour et al. 2015; Hilborn et al. 2003; Schindler et al. 2010). At larger temporal and spatial scales, genetic population structure in large lakes has mostly been affected by post‐glacial recolonization (Bernatchez and Wilson 1998) and isolation‐by‐distance (e.g., Euclide, Kuhl, et al. 2024). However, at finer scales, local adaptation to heterogeneous environments (e.g., Shi et al. 2022), spatial segregation during the feeding season (e.g., Dupont et al. 2007; Potvin and Bernatchez 2001), philopatry to natal spawning grounds (e.g., Wilson et al. 2016) or tributaries (e.g., Homola et al. 2010, 2012) and even pelagic larval dispersal patterns (Schraidt et al. 2023) have been shown to shape intra‐lacustrine genetic structure of freshwater fish populations in large lakes.

In vast open systems (e.g., marine systems; Gagnaire et al. 2015), the combination of relatively elevated levels of gene flow and putatively low genetic drift (Nielsen et al. 2009) can limit genetic differentiation to regions of the genome that are involved in local adaptation (Kawecki and Ebert 2004) and lead to cryptic population structure that is difficult to disentangle using a limited number of neutral markers (Gagnaire et al. 2015; Waples 1998). In this context, high‐density genomic data like whole‐genome sequencing can be essential to disentangle complex population structure (Fuentes‐Pardo and Ruzzante 2017; Hohenlohe et al. 2021), as genome‐wide scans enable the detection of highly localized regions of differentiation that might otherwise go unnoticed (Benestan 2020; Bernatchez et al. 2017; e.g., Akopyan et al. 2025; Bradbury et al. 2013; Cayuela et al. 2020; Nikolic et al. 2023; Shi et al. 2023). These regions are often characterized by the tight clustering of loci in small, often low‐recombining (Samuk et al. 2017) sections of the genome. Their emergence has been attributed either to divergence hitchhiking, in which gene flow is reduced in sites that are physically linked to genes under strong divergent selection (Nosil et al. 2009; Via 2012) or through the clustering of locally adapted alleles in structural variants such as chromosomal inversions (Kirkpatrick and Barton 2006; Yeaman 2013), which can cluster neighboring co‐adapted alleles and maintain them in the face of gene flow by suppressing recombination in heterokaryotypes (Berdan et al. 2023; Hoffmann and Rieseberg 2008; Tigano and Friesen 2016; Wellenreuther and Bernatchez 2018).

Recently, commercially harvested fish populations from large lakes have benefited from a genomic reassessment of population structure, which often reveals fine‐scale population structure that was previously undetected using neutral genetic markers. For instance, high‐density genomic data enabled the detection of putative chromosomal inversions associated with fine‐scale differentiation of Lake Whitefish ( *Coregonus clupeaformis* ) populations in Lake Michigan (Shi et al. 2022) and Lake Erie (Euclide et al. 2022). Similarly, a putative chromosomal inversion seems involved in lake‐wide local adaptation in Walleye ( *Sander vitreus* ) from three large Manitoban lakes despite elevated levels of gene flow (Thorstensen et al. 2022).

While genetic structure analyses have been conducted for most commercially harvested fish species in the Laurentian Great Lakes, fewer have been conducted in the northern Great Lakes of Canada (but see Bernos et al. 2024; Elmer et al. 2008; Turgeon and Bourret 2013; Turgeon et al. 2016; Wiens et al. 2021). For instance, in Great Slave Lake, the genetic structure of Lake Whitefish populations, which have supported the largest commercial fisheries in the Western Arctic since the mid‐1940s (Department of Fisheries and Oceans [DFO] 2015; MacKenzie et al. 2022; Rawson 1947, 1949), has never been assessed. In this large subarctic lake (28,570 km^2^), multiple factors could contribute to divergence among Lake Whitefish populations. First, in large lakes, Lake Whitefish can demonstrate natal spawning site fidelity (Ebener et al. 2010, 2021; Ryther et al. 2024; Walker et al. 1993) and spatial niche segregation (Beech et al. 2024) that can result in temporally stable genetic differentiation (Nathan et al. 2016). In such cases, gene flow is expected to be reduced between distant populations, leading to isolation‐by‐distance (Wright 1943). Spawning site fidelity could be especially strong in adfluvial populations, which spawn in tributaries after feeding in Great Slave Lake during the summer and that are believed to occur in the Slave (McLeod et al. 1985; Tallman 1996; Tallman et al. 2005; Tripp et al. 1981), Little Buffalo (Roberge et al. 1985) and Yellowknife (Miller et al. 2025) rivers. Finally, the environmental heterogeneity of Great Slave Lake could have led to the emergence and maintenance of locally adapted populations. Indeed, inflow from tributaries combined with depth variations shape regional differences in limnology in Great Slave Lake, especially between the two main basins of the lake. In the Main Basin (fisheries management areas IW, IE, II, III, IV and V), important inflow from the Slave River influences turbidity, water surface temperature and dissolved oxygen levels (Zhu et al. 2017), which have cascading effects on the planktonic, benthic and fish communities (Evans 2000; Fee et al. 1985; Rawson 1950, 1951, 1953, 1956; Zhu et al. 2017). In contrast, the Slave River has less influence on the deep waters of the East Arm. This region is consequently less productive and exhibits comparatively homogeneous ultraoligotrophic limnological characteristics (Evans 2000; Rawson 1950).

In this study, we used low‐coverage whole‐genome resequencing to describe the genetic structure of Lake Whitefish populations in Great Slave Lake. We first documented patterns of genetic differentiation among sampling locations to identify genetically differentiated populations. We then investigated regions of elevated genetic differentiation using genome‐wide scans to detect outlier regions putatively under selection as well as putative structural variants. We hypothesized that Great Slave Lake would harbour multiple genetically differentiated populations of Lake Whitefish and that structural variants could play a role in maintaining genetic differentiation between putatively locally adapted populations.

## 2 Material and Methods

### 2.1 Sampling

**FIGURE 1:**
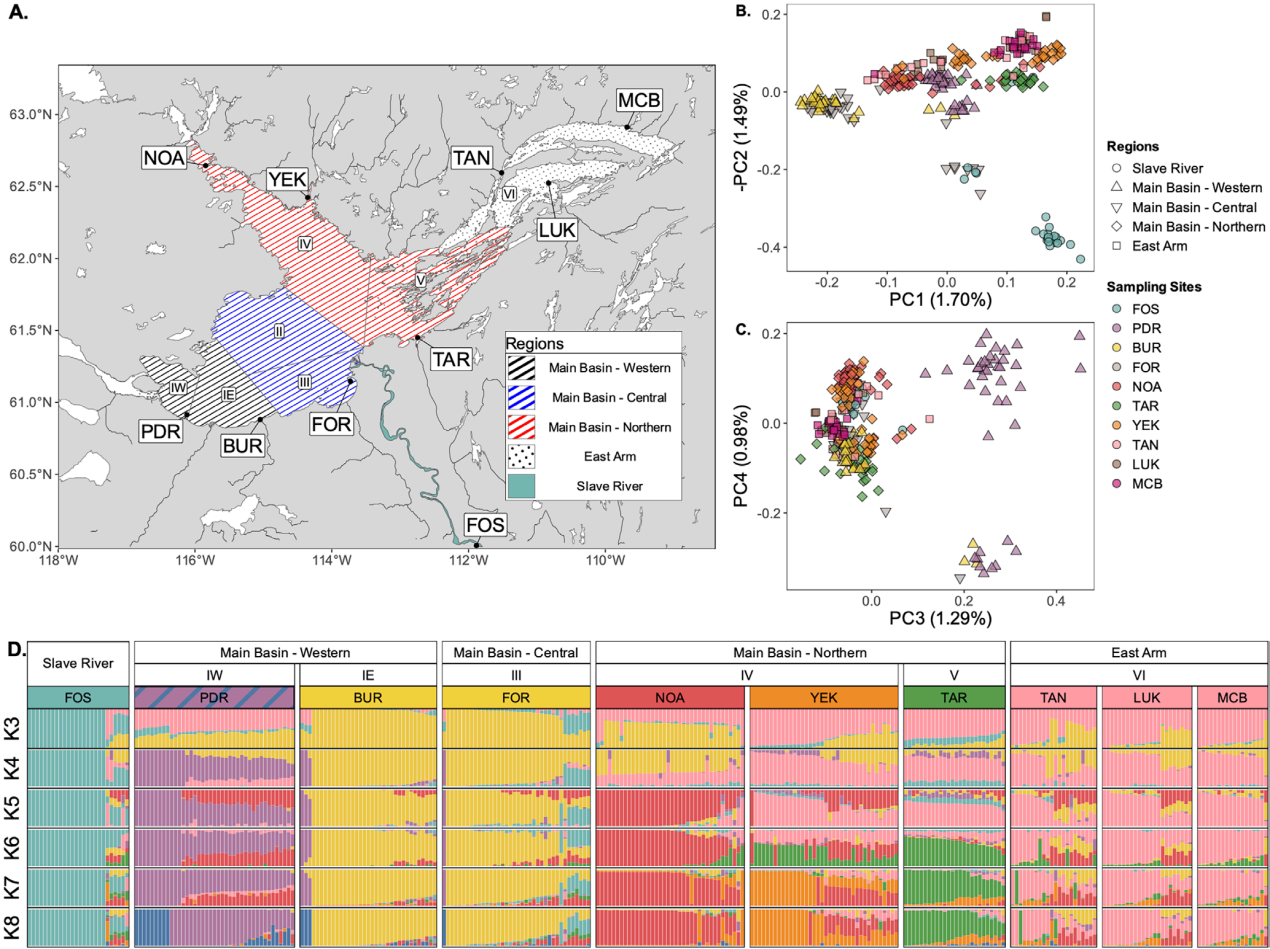
Genetic population structure analyses of Lake Whitefish from ten sampling sites in the Great Slave Lake area. Sampling sites: FOS, Fort Smith; PDR, Pointe de Roche; BUR, Buffalo River; FOR, Fort Resolution; NOA, North Arm; YEK, Yellowknife; TAR, Taltson River; LUK, Łutsël K'é; TAN, Taltheilei Narrows; MCB, McLeod Bay. (A) Map of Great Slave Lake showing the sampling sites in three regions: Main Basin, East Arm, and Slave River. The Main Basin is split into three subregions: Western, Central, and Northern Basins. Commercial fishing management areas (FMA) are identified by roman numerals (IE, IW, II, III, IV, V, VI). (B) PCA of the first two principal components obtained using PCAngsd on the LD‐pruned SNP dataset (181,603 SNPs), individuals are colored by sampling sites, while shapes represent regions. (C) PCA of the third and fourth principal components obtained using PCAngsd on the LD‐pruned SNP dataset (181,603 SNPs), individuals are colored by sampling sites while shapes represent regions. (D) Individual admixture proportions at *K* = 3, *K* = 4, *K* = 5, *K* = 6, *K* = 7, and *K* = 8 by sampling site, grouped by region and management areas.

Lake Whitefish were collected at 10 sampling sites selected across the lake based on the presence of spawning aggregates and/or their proximity to major river systems (Figure 1A, Table 1). Fin clips from a total of 337 Lake Whitefish were taken for genetic analyses at nine putative spawning locations in shallow nearshore areas of Great Slave Lake itself, and one spawning location (Fort Smith) in the Slave River, its main tributary. Adult adfluvial Lake Whitefish have been suggested to return to Fort Smith each fall to spawn after feeding in Great Slave Lake during the summer (McLeod et al. 1985; Tallman 1996; Tallman et al. 2005; Tripp et al. 1981). Lake Whitefish were collected either by local Indigenous partners, local commercial fishers, or by DFO biologists using large mesh size (≥ 51 mm) gillnets, which should have mostly targeted mature fish. Whitefish in Great Slave Lake were all collected between September 18th and October 21st in 2019 and 2020. Putative adfluvial whitefish, sampled in Fort Smith, were collected in the last week of August and the first week of September, when the pre‐spawning adults begin to appear at the base of the rapids.

**TABLE 1:** Summary of sample sizes and genomic diversity estimates of Lake Whitefish from ten sampling sites around Great Slave Lake.

Sampling site	Code	Latitude	Longitude	Region	Genetic cluster	N_sampled_	N_final_	Het (kb^−1^)	θ_W (1000)_	θ_π (1000)_	Tajima's D
Fort Smith	FOS	60.0055	−111.8849	Slave River	Fort Smith	26	26	2.66 ± 0.35	4.16 ± 0.87	1.77 ± 1.10	−2.14 ± 0.55
Pointe de Roche	PDR	60.9142	−116.1183	Main Basin‐ Western	Pointe de Roche	41	32	2.80 ± 0.40	4.28 ± 0.97	1.82 ± 1.18	−2.10 ± 0.56
—	—	—	—	Main Basin—Western	Hay River	—	13	2.62 ± 0.25	3.00 ± 0.96	1.84 ± 1.14	−1.66 ± 0.61
Buffalo River	BUR	60.8807	−115.0457	Main Basin—Western	South Shore	40	32	2.86 ± 0.39	4.91 ± 0.92	1.81 ± 1.13	−2.28 ± 0.50
Fort Resolution	FOR	61.1457	−113.7258	Main Basin—Central	South Shore	39	37	2.55 ± 0.27	4.15 ± 0.87	1.65 ± 1.12	−2.17 ± 0.56
North Arm	NOA	62.6456	−115.8442	Main Basin—Northern	North Arm	39	37	2.47 ± 0.26	3.93 ± 0.87	1.62 ± 1.14	−2.12 ± 0.59
Yellowknife	YEK	62.4231	−114.3516	Main Basin—Northern	Yellowknife	39	39	2.49 ± 0.17	4.06 ± 0.86	1.60 ± 1.13	−2.16 ± 0.58
Taltson River	TAR	61.4506	−112.7447	Main Basin—Northern	Taltson River	27	26	2.51 ± 0.20	3.44 ± 0.94	1.68 ± 1.16	−1.95 ± 0.63
Taltheilei Narrows	TAN	62.5951	−111.5162	East Arm	East Arm	36	21	3.60 ± 1.44	6.43 ± 1.14	2.44 ± 1.10	−2.33 ± 0.39
Łutsël *K*'é	LUK	62.5230	−110.8299	East Arm	East Arm	27	24	2.51 ± 0.22	3.31 ± 0.94	1.65 ± 1.15	−1.92 ± 0.63
McLeod Bay	MCB	62.9131	−109.6823	East Arm	East Arm	23	18	4.19 ± 1.54	7.77 ± 1.10	2.97 ± 1.14	−2.37 ± 0.35

*Note:* Individuals from the Hay River genetic cluster were sampled in Pointe de Roche (9), Buffalo River (3), and Fort Resolution (1). Values for diversity estimates are reported as mean ± SD.

Abbreviations: *N*_Sampled_, number of whitefish sampled at each sampling sites; *N*_final_, number of whitefish from each sampling sites/genetic clusters after filtering and reclassifying F0 migrants; Het (kb^−1^), Mean individual autosomal heterozygosity (number of heterozygous site per kb); θ_W (1000)_, mean Watterson's estimate per 1000 sites in 25 kb windows (5 kb overlap); θ_π (1000)_, mean nucleotide diversity per 1000 sites in 25 kb windows (5 kb overlap); Tajima's D, mean Tajima's D estimate in 25 kb windows (5 kb overlap).

### 2.2 DNA Extraction and Sequencing

Genomic DNA was first extracted from fin clips or muscle samples preserved in 95% ethanol kept at −20°C, using the NucleoMag Tissue Kit (Macherey‐Nagel) with an RNAse A (Qiagen) treatment. After extraction, DNA concentrations were assessed using the AccuClear Ultra High Sensitivity DsDNA Quantitation Kit (Biotium) and normalized to 2 ng/μL after a first cleaning step using Axygen magnetic beads. Nextera libraries for low‐coverage whole‐genome resequencing were prepared using 2 ng/μL DNA extracts that were randomly redistributed on four 96‐well library plates, following a protocol described by Baym et al. (2015) and Therkildsen and Palumbi (2017) and modified according to Mérot et al. (2021). First, a tagmentation step (Nextera kit, Illumina) was used to fragment DNA and add partial adapters. Then, a two‐step PCR was performed using the KAPA Library Amplification Kit (Roche). During the first PCR step, individual barcodes were added to the fragments to identify reads. During the second PCR step, Illumina adapters (i5‐i7) were added to the fragments. Finally, Axygen magnetic beads were used for a cleanup step to keep only DNA fragments between 400 and 1000 bp in size. Equimolar amounts of 96 libraries were then combined in four separate pools after assessing their concentration using the AccuClear Ultra High Sensitivity dsDNA Quantitation Kit (Biotium). The fragment size distribution of a random sample of the library pools was assessed using the Agilent BioAnalyzer (Agilient) to verify if the mean insert size distribution was between 500 and 700 bp.

Samples were sequenced using 150 bp paired‐end reads on an Illumina Novaseq 6000 S4 at McGill Génome Québec Innovation Center, with a target sequencing depth of 2X. Raw reads were processed with a pipeline for Whole‐Genome Sequencing (WGS) sample preparation available at https://github.com/enormandeau/wgs_sample_preparation, inspired by Therkildsen and Palumbi (2017). This pipeline used FastP v0.20.1 (default settings, Chen et al. 2018) to trim and filter reads for quality, then used BWA‐MEM v0.7.17‐r1188 (default settings, Li and Durbin 2009) to align them to the reference genome. In this case, reads were aligned to an older version of the currently available reference genome for Lake Whitefish ( *Coregonus clupeaformis* ) (Assembly ASM1839867v1; GCA_018398675.1; 01/21; Mérot et al. 2023). After alignment, reads were filtered to keep those with a mapping quality over 10 using Samtools v1.8 (Danecek et al. 2021), duplicate reads were removed using MarkDuplicates (PicardTools v1.119), indels were realigned using IndelRealigner from GATK 3.5 (McKenna et al. 2010) and overlapping read ends were soft‐clipped using clipOverlap in bamUtil v1.0.15 (Breese and Liu 2013).

### 2.3 SNP Calling and Filtering

As our targeted sequencing depth (~2X) was too shallow to accurately genotype SNPs, we used a probabilistic framework for this study (Lou et al. 2021). As such, SNP calling and filtering were done in ANGSD v.0.931 (Korneliussen et al. 2014) using a pipeline developed by Mérot et al. (2021) (https://github.com/clairemerot/angsd_pipeline). First, ANGSD v.0.931 was used to call the SNPs (*−remove_bads 1, −minMapQ 30, −minQ 20, −skipTriallelic 1*) in our sequencing data and calculate their genotype likelihoods (GL, *−GL 2*) and minor allelic frequency (MAF, *−doMaf* 1). SNPs were kept for analysis only if their MAF was over 0.05 and if at least 75% of the samples presented depth of coverage of at least 1X. A maximum average depth of 10X was also set for the called SNPs.

Then, NGSparalog (Linderoth 2018; https://github.com/tplinderoth/ngsParalog) was run to filter out SNPs deviating from expected patterns of heterozygosity and allelic ratio due to the mismapping of sequences on the reference. These deviant SNPs, widespread in salmonid genomes, are often the result of residual tetrasomy or repeated elements and can impact population genomics analyses (Dallaire et al. 2023). In brief, we used the calcLR script provided in NGSparalog to calculate the likelihood ratio of mismapping reads (minQ = 20) covering each site. We then removed sites that were significantly likely to be missmapped using a Benjamini‐Hochberg adjusted *p* value < 0.001. To produce a subset of unlinked SNPs for principal component (PCA) and admixture analyses, we used ngsLD (Fox et al. 2019) to estimate linkage disequilibrium between SNPs, then removed highly linked SNPs until no SNP pair within 200 Kb had a *r* ^*2*^ above 0.075. The thresholds used for LD‐pruning were determined by estimating LD‐decay.

### 2.4 Sample Filtering

Because some population structure analyses can be highly affected by missing data (e.g., NGSadmix; Skotte et al. 2013), samples showing high levels of missing data (above 95th percentile, i.e., 38% of SNPs missing GL) were removed from the dataset. The level of missingness per sample was calculated using a java script (gprobssamplemissing.jar) developed by Browning (2013) and available on the Beagle utilities webpage:https://faculty.washington.edu/browning/beagle_utilities/utilities.html. Mean sequencing depth per sample was also calculated using mosdepth v.0.3.6 (Pedersen and Quinlan 2018). Putatively duplicated individuals were also identified and removed from the analysis (Supporting Information).

### 2.5 Population Genomics Analyses

#### 2.5.1 Population Structure

To visualize population genetic structure, PCAngsd v.1.10 (Meisner and Albrechtsen 2018) was used to conduct PCA analyses using the 181,603 LD‐pruned SNP dataset. The R package *vegan v.2.6‐4* (Oksanen et al. 2022) was used to generate the PCA from the covariance matrix outputted by PCAngsd. We assessed which PC axes were statistically relevant visually using a scree‐plot.

Using this same set of LD‐pruned SNPs, *NGSadmix* (Skotte et al. 2013) was used to estimate the admixture proportions of each sample assuming a number of genetic groups ranging from *K* = 2 to 15. Other parameters were set to default (Maximum number of EM iterations: *‐maxiter* = 2000, Tolerance for convergence: *‐tol* = 1 × 10^−5^). For each value of *K*, the analysis was repeated 50 times. We then ran Clumppling v.1.0.2 to align multiple replicate solutions of unsupervised clustering analyses (default settings; Liu et al. 2024; https://github.com/PopGenClustering/Clumppling) and obtain different modes for each *K*. For visualization purposes, we selected the averaged run of the most statistically supported mode for each value of *K* (see mode stats in Table S1). To statistically assess the optimal number of genetic clusters, we used *StructureSelector* (Li and Liu 2018), a web‐based software that estimates the best *K* using the four Puechmaille estimators (MedMedK, MedMeanK, MaxMedK, MaxMeanK; Puechmaille 2016). These estimators were used as they alleviate statistical problems related to uneven sample sizes between hierarchical levels of population structure (Puechmaille 2016). We used threshold values of 0.5, 0.6, 0.7, and 0.8 for each estimator, as suggested by Puechmaille (2016). The software *evalAdmix* (Garcia‐Erill and Albrechtsen 2020) was also used to assess the statistical fit of the admixture model at different values of *K*. For *evalAdmix*, for each *K*, we used the run that had the highest maximum log‐likelihood value according to *NGSadmix*. Individuals who had a membership probability of *q* > 0.99 to a genetic cluster different from the dominant genetic clusters of their sampling location were assumed to be F0 migrants and were reassigned.

#### 2.5.2 Genetic Differentiation

The set of SNPs filtered for deviant SNPs (1,432,187 SNPs) was used to calculate pairwise F_ST_ values between sampling sites. First, site allele frequencies (SAF) were estimated for each sampling location using the *‐dosaf* 1 flag in ANGSD. The flags ‐*doMajorMinor* 3 and *‐sites* were used to polarize alleles manually. Then, *realSFS* was used to compute a two‐dimensional site frequency spectrum (2D‐SFS) for each sampling site pair. F_ST_ values were calculated using the *realSFS fst index*, *print*, *stats*, and *stats2* functions. We used Hudson's F_ST_ (*‐whichFST* 1 flag in *realSFS*), as it is less sensitive to uneven sampling sizes (Bhatia et al. 2013).

We also calculated pairwise allelic frequency differences (AFD) between populations, as this differentiation metric is more linear, less sensitive to sample size differences, and has been suggested to be more adapted to systems with weak genetic differentiation (Berner 2019). Pairwise allele frequency difference (AFD) values were calculated by averaging the absolute difference between minor allele frequencies in populations, generated using the *‐doMaf* 1 *‐doMajorMinor* 3 flags in ANGSD v.0.931.

Isolation‐by‐distance (IBD; Wright 1943) was evaluated by correlating pairwise linearized‐F_ST_ (F_ST_/(1 – F_ST_)) and AFD to in‐water distance between sampling sites. The least‐cost in‐water distances between sampling sites were calculated using the *costDistance* function in the R package *gdistance v.1.6.4* (van Etten 2017) by setting an infinite resistance to land and a resistance of one to water, as described by Shi et al. (2022). Mantel tests (Mantel 1967) with 9999 permutations were conducted in the *vegan v.2.6‐4* package (Oksanen et al. 2022) in R to test the statistical significance of IBD.

#### 2.5.3 Genomic Diversity Estimations

We estimated genomic diversity in populations with Watterson's estimator (Θ_W_), nucleotide diversity (Θπ) and individual autosomal heterozygosity. The reference genome used for genomic diversity measures was previously masked to remove deviant SNPs (i.e., those previously filtered using NGSparalog). Briefly, the masking step was done by first extracting 300 bp regions centred around deviant SNPs using the *GenomicRanges* package (Lawrence et al. 2013) in R and by hard‐masking these regions in the reference genome using *bedtools maskfasta* (Quinlan and Hall 2010).

Individual autosomal heterozygosity was calculated by first computing a per‐sample SAF using ANGSD v.0.937 with the *‐doSaf* 5 and *‐doMajorMinor* 5 arguments to manually set the major allele to the reference genome of *C. clupeaformis* masked for deviant SNPs. Then, the resulting individual SAF was used to produce a 1D‐SFS using *winsfs* (Rasmussen et al. 2022), and the autosomal heterozygosity was calculated by dividing the number of heterozygous sites (SAF = 1) by the total number of sites.

For Θ estimations, population SAF and 1D‐SFS were generated as described for individual heterozygosity. Thetas were then calculated using ANGSD's *realsfs saf2theta* and *thetaStat do_stat* functions. We estimated per‐site Θ in windows of 25Kb (step of 5Kb) and excluded windows with less than 1000 sites in any population. To minimize biases related to sample size differences, Θ estimations were done on a random subsample of 26 individuals per site, except for Hay River, which had only 13 individuals.

#### 2.5.4 Genome Scan

A genome scan was performed using an extended version of FastPCA (Galinsky et al. 2016) implemented in PCangsd (*−selection*; Meisner et al. 2021). FastPCA is a genome scan method based on principal component analysis (PCA) that infers selection by identifying SNPs that differ significantly from the expected null distribution of allelic frequencies under genetic drift for each relevant PC axis, which should follow a chi‐square (1 d.o.f.) distribution under the null hypothesis of no selection (Galinsky et al. 2016). In our case, selection scans using FastPCA were done on the first four PC axes, as identified using a scree plot. To correct for false‐discovery rate, *p*‐values obtained from FastPCA were converted to *q*‐values using the *qvalue (v. 2.38.0)* package in R (Storey et al. 2024). SNPs were considered outliers if *q* < 0.01. Candidate regions of elevated genetic differentiation were identified by splitting our dataset into non‐overlapping windows of 1000 SNPs and identifying windows with > 10 FastPCA outliers. Outlier windows separated by less than 10 windows were combined into candidate regions.

#### 2.5.5 Putative Structural Variant Detection and Characterization

To identify potential structural variants, a local PCA approach was used to detect large regions displaying distinct genetic variation following scripts by Mérot et al. (2021) (*10_pca_by_window*; https://github.com/clairemerot/angsd_pipeline). First, PCAs were done on non‐overlapping windows of 100 SNPS on each chromosome in parallel. The R package *lostruct* (Li and Ralph 2019) was then used to measure the similarity between local PCAs on each chromosome independently using Euclidean distances on the first 4PC axes. Using a similar approach to Dallaire et al. (2025), results from *lostruct* were mapped on 5 MDS axes and outlier windows were determined as those having MDS values over or under 3 standard deviations from the mean. Outlier windows were merged into clusters if they were within 20 windows of each other. Only clusters containing 10 outlier windows or more were kept for the rest of the analyses. Putative inversions were identified visually as candidate regions showing three distinct clusters in local PCA, a common pattern for inversions (e.g., Huang et al. 2020; Lotterhos 2019; Shi et al. 2023). Individuals were assigned to a putative genotype by *K*‐means clustering implemented in the ClusterR package (Mouselimis 2024), with the group located near the middle of the PC1 axis assigned as the “AB” heterogeneous genotype and the most common homozygous group identified as “AA”. For some putative inversions, *K*‐means could not reassign the group effectively due to skewed cluster shapes. For these inversions (e.g., on chromosome 1), *K*‐means was used to classify individuals into more than three groups, then these groups were visually classified into three groups based on their position on PC1. After assigning individuals to genotypes in the local PCA of putative inversions, we estimated the heterozygosity in all individuals to verify if the putative heterogeneous individuals displayed elevated SNP heterozygosity (as seen in Huang et al. 2020). This was done with a similar approach described above for autosomal heterozygosity, but limiting the analysis to SNPs in each candidate region. A Wilcoxon test was used to test whether heterozygosity was elevated in this group compared to the two other groups. We also calculated the frequency of each putative inversion genotype at each sampling location and plotted them on a map using the R package *mapmixture* (Jenkins 2024).

We also tested whether LD levels were elevated inside putative inversions overlapping with regions of elevated genetic differentiation detected through the genome scan. To do this, we visualized LD patterns in all individuals in a heatmap by computing *R* ^2^ values inside the putative inversion and surrounding regions of the same size using *ngsLD*. To refine inversion boundaries, we used a method described in Euclide, Larson, et al. (2024) where the starting position of the inversion is defined as the SNP that is tightly linked (top 5% of LD in the given chromosome, max_dist = 200Kb) to the highest number of downstream SNPs in the 5′ direction and the end position is defined as the SNP that is tightly linked to the highest number of upstream SNPs in the 3′ direction. We also used a method described in Euclide, Larson, et al. (2024) to assess if LD was more elevated in the putative inversions compared to other regions on the same chromosome. To do so, we calculated the mean R^2^ value among SNPs within putative inversions to the mean *R* ^2^ value in 1000 random windows of the same size on the same chromosome. Statistical significance was then assessed by calculating a one‐tailed *p* value (α = 0.05) with a *Z*‐test.

Finally, we investigated the presence of genes in regions of elevated genetic differentiation and putative inversions by first reporting the coordinates of the candidate regions on the published reference genome of the Lake Whitefish (normal type; accession GCA_018398675.1; ASM1839867v1) using DGENIES (Cabanettes and Klopp 2018), then computing overlaps between these regions and known genes in the ASM1839867v1 assembly using *bedtools window* with parameter‐w 0 (version 2.30.0; Quinlan and Hall 2010). For this purpose, a custom annotation table was built using a workflow (https://github.com/FlorentSylvestre/GO_analysis) adapted from the pipeline GAWN v0.3.5 ([https://github.com/enormandeau/gawn]). Briefly, this pipeline retrieves GO terms associated with the genes reported in the ASM1839867v1 assembly General Feature Format (GFF) file by mapping transcripts against the Swissprot 1.2 database (UniProt Consortium 2025) using blast 2.12.0 (Camacho et al. 2009). Using this custom annotation table, we then investigated gene ontology term enrichment with *goatools* 1.4.12 (Klopfenstein et al. 2018) using the different sets of candidate regions and the go‐basic database version 1.2 (2025‐07‐22; https://release.geneontology.org/2025‐07‐22/ontology/go‐basic.obo). Only enriched terms with a corrected *p*‐value (Benjamini and Hochberg 1995) under 0.05 were considered significant.

## 3 Results

### 3.1 Sequencing, SNP Calling & Filtering

After low‐coverage resequencing of 337 Lake Whitefish from 10 sampling sites, we obtained a median mean depth of 1.51X (0.83–3.3X, Figure S1) of aligned and filtered sequences per sample. A total of 4,627,132 SNPs were kept after filtering for minor allele frequency (> 0.05) and maximum depth (< 10X) and keeping only SNPs with at least 1X in 75% of individuals. Out of those SNPs, 1,432,138 SNPs were considered non‐deviant after filtering for putative paralogs. After LD‐pruning, only 181,603 SNPs were kept for PCA and admixture analyses. Out of our 337 samples, 28 had elevated levels of missing data (> 38%) and were removed from the dataset. Additionally, four individuals were removed as they were potentially duplicates of another individual, leaving a total of 305 Lake Whitefish for downstream analyses. Sample sizes after filtration for each sampling site varied between 18 and 39 fish (Table 1).

### 3.2 Population Structure Analyses

After computing PCAs from the LD‐pruned SNP dataset, it was determined that only the first four PC axes were statistically significant using a scree‐plot (Figure S4). From these four PC axes, three main patterns emerged. First, on PC1 (1.70% explained; Figure 1B), individuals sampled at the BUR (in grey) and FOR (in yellow) sites in the Western and Central Basin were clearly distinct from individuals from other sampling locations in Great Slave Lake. Still on PC1, individuals from other Western (PDR) and Northern Basin (NOA, TAR) sites formed relatively distinct clusters, while some, like those caught in the East Arm (TAN, LUK, MCB), heavily overlapped. On PC2 (1.49%; Figure 1B), samples from the FOS site (in turquoise), in the Slave River, differed from the Great Slave Lake populations. Some individuals sampled in FOR and FOS also formed a distinct group located in the middle of this axis. On PC3 (1.29%; Figure 1C), samples from the PDR site, in the Western Basin, differed from those sampled at other locations. On PC4 (0.98%; Figure 1C), samples from PDR split into two distinct clusters, suggesting that Whitefish from two discrete populations were sampled at that location.

Admixture proportions between *K* = 2 and *K* = 8 suggested a hierarchical population genetic structure among sampling sites. Beginning at *K* = 3 (Figure 1D), samples from the FOS site, in the Slave River, showed a distinct genetic ancestry from those sampled in Great Slave Lake. From *K* = 4 to *K* = 8 (Figure 1D), sampling sites in the two main regions of Great Slave Lake (Main Basin and East Arm) split into multiple discrete genetic clusters. At *K* = 4 (Figure 1D), samples from the PDR site (in purple), located in the Western Basin (FMA IW) split from those sampled at the BUR and FOR sites (in yellow), located in the Southern shore of fisheries management areas IE and III, in the Western and Central Basin. Then, sampling sites located in the Northern Basin (FMAs IV and V) split into multiple distinct genetic clusters, with individuals sampled at the NOA (in red), TAR (in green) and YEK (in orange) sites splitting from other sampling locations at *K* = 5, *K* = 6 and *K* = 7, respectively (Figure 1D). Finally, at *K* = 8 (Figure 1D), some individuals from the PDR, BUR and FOR sampling sites form a distinct genetic cluster (in blue). Using different thresholds (0.5–0.8), the Puechmaille estimators (*MedMedK*, *MedMeanK*, *MaxMedK*, *MaxMeanK*) found best *K* values ranging between *K* = 4 and *K* = 8 (Table S1.1). According to *evalAdmix* the correlation between residuals indicated insufficient number of *K* clusters when *K* < 7 (Figure S2). Above *K* = 8, clusters of samples were not clearly associated with geography and were less stable across iterations according to *Clumppling* (Table S1.2, Figure S3.2). Therefore, *K* = 8 (Figure 1D) was chosen as the most probable number of genetic clusters. Thus, after the admixture analysis, individuals from the TAN, LUK and MCB sampling sites were considered part of a single genetic cluster named East Arm (in pink, Figure 1D). Similarly, individuals from the BUR and FOR sampling sites were considered part of a single genetic cluster named South Shore (in yellow, Figure 1D). Individuals sampled at the PDR, BUR and FOR sites that demonstrated a similar distinct ancestry (in blue, Figure 1D) were grouped as a distinct genetic cluster named Hay River (HAY). All the other sampling sites (FOS, PDR, NOA, YEK and TAR) formed their own distinct genetic clusters at *K* = 8. One individual from the NOA site was reassigned to the nearby Yellowknife genetic cluster as it had a pure genetic ancestry corresponding to the dominant genetic cluster at the YEK sampling site (Q = 1). At *K* = 8, putatively admixed individuals (Q = < 0.8) were present at all sampling sites, but particularly prevalent (> 50%) in sites from the Northern Basin (YEK, TAR) and East Arm (TAN, LUK, MCB) (Table S1.3).

### 3.3 Genetic Differentiation & Isolation‐By‐Distance

**FIGURE 2:**
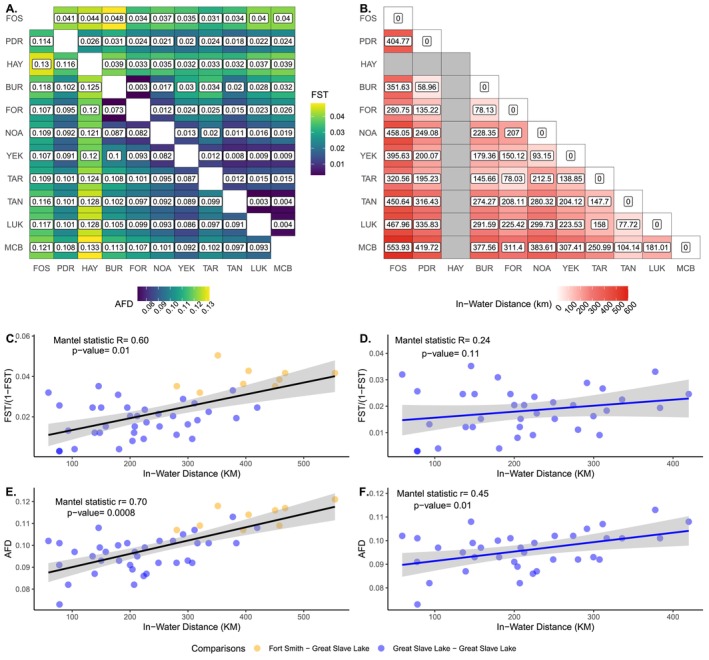
Population differentiation and in‐water distances patterns between ten sampling sites in Great Slave Lake and Slave River. Sampling sites: FOS, Fort Smith; PDR, Pointe de Roche; BUR, Buffalo River; FOR, Fort Resolution; NOA, North Arm; YEK, Yellowknife; TAR, Taltson River; LUK, Łutsël *K*'é; TAN, Taltheilei Narrows; MCB, McLeod Bay. (A) Heatmap of pairwise F_ST_ (upper triangle) and pairwise AFD (lower triangle) values between ten sampling sites in Great Slave Lake and Slave River calculated on the full set of 1,432,138 SNPs. The Hay River (HAY) genetic cluster, composed of individuals sampled at the PDR, BUR & FOR sampling sites was considered as a distinct group in this heatmap. (B) Heatmap of in‐water distance (in km) between ten sampling sites in Great Slave Lake and Slave River. (C) Isolation‐by‐distance plot showing the correlation between pairwise linearized F_ST_ (F_ST_/(1 – F_ST_)) and in‐water distance between sampling sites in Great Slave Lake and Slave River. (D) Isolation by distance plot showing the correlation between pairwise AFD values and in‐water distance between sampling sites in Great Slave Lake and Slave River. (E) Isolation‐by‐distance plot showing the correlation between pairwise linearized F_ST_ (F_ST_/(1 – F_ST_)) and in‐water distance between sampling sites in Great Slave Lake only. (F) Isolation‐by‐distance plot showing the correlation between pairwise AFD and in‐water distance between sampling sites in Great Slave Lake only.

Pairwise genetic differentiation between sampling sites was generally low, with pairwise F_ST_ values ranging from 0.003 to 0.048 and pairwise AFD values ranging from 0.073 to 0.133 (Figure 2A). Genetic differentiation patterns evaluated using pairwise F_ST_ values were consistent with what was observed in the admixture and PCA analyses. The BUR and FOR sampling sites, which were grouped as one genetic cluster (South Shore) had a pairwise F_ST_ of only 0.003. Similarly, the LUK, TAN and MCB sampling sites, which were grouped as one genetic cluster (East Arm), had pairwise F_ST_ values of 0.003–0.004. The FOS sampling site, sampled in the Slave River, 280 km upstream from the closest sampling site in the lake, was the most genetically differentiated (pairwise F_ST_ = 0.031–0.048; Figure 2A). Genetic differentiation was generally low between sampling sites located east of the Slave River, in the Northern Basin and the East Arm (NOA, YEK, TAR, TAN, LUK, MCB), with pairwise F_ST_ values between 0.003 and 0.02. The YEK sampling site, which formed a distinct genetic cluster according to the admixture analyses, was only weakly differentiated (pairwise F_ST_ = 0.008–0.009) from sampling sites from the East Arm (TAN, LUK, MCB). Individuals from the two distinct genetic clusters sampled at the PDR sampling site according to the admixture analysis, Pointe de Roche and Hay River, were genetically differentiated from each other (pairwise F_ST_ = 0.026), and from the nearby sampling locations of BUR (pairwise F_ST_ = 0.031–0.039) and FOR (pairwise F_ST_ = 0.024–0.033). Surprisingly, the sampling site located at the northern extremity of the Northern Basin, NOA, was as genetically differentiated from the nearby YEK sampling site (pairwise F_ST_ = 0.012, in‐water distance = 93.15 km) as it was from further sampling sites located in the Central Basin (e.g., FOR; pairwise F_ST_ = 0.012, in‐water distance = 207 km) and East Arm (e.g., TAN; pairwise F_ST_ = 0.01, in‐water distance = 280.32 km).

Genetic differentiation patterns evaluated using pairwise AFD values between sampling sites mostly mirrored those using pairwise F_ST_. The YEK sampling site had very low pairwise AFD values with some East Arm sampling sites (LUK, TAN, MCB; 0.086–0.092). These low pairwise AFD values were similar to those between East Arm sampling sites (0.091–0.097), which were considered part of the same genetic cluster according to the NGSadmix analysis.

Mantel tests revealed a statistically significant correlation between genetic and in‐water distances among sampling sites, consistent with isolation‐by‐distance (Figure 2C,E). The correlation was stronger when using pairwise AFD (Mantel *R* = 0.70, *p* value = 8 × 10^−4^) than when using pairwise linearized F_ST_ (Mantel *R* = 0.60, *p* value = 0.01). However, when excluding the Slave River sampling site, the correlation between genetic and in‐water distances was weaker (Figure 2D,F, pairwise linearized F_ST_: Mantel *R* = 0.24; pairwise AFD: Mantel *R* = 0.45), and was only supported statistically when using AFD (*p* value = 0.01).

### 3.4 Genomic Diversity Estimations

Individual autosomal heterozygosity varied significantly between sampling locations (Table 1; Kruskal–Wallis test *p* = 7.7 × 10^−14^). Individuals from the sampling locations of TAN and MCB demonstrated relatively high and variable levels of autosomal heterozygosity compared to the other sampling locations. Similarly, TAN and MCB individuals had relatively high mean per‐site Θ_W_ and per‐site Θπ values (Table 1). Individuals from the Hay River (HAY) genetic cluster had smaller mean per‐site Θ_W_ estimates than individuals from other sampling locations. Tajima's D values in each sampling site were all negative, with smaller mean estimates in the TAN and MCB sampling sites and a higher mean estimate in the Hay River (HAY) genetic cluster (Table 1).

### 3.5 Genome Scans & Putative Structural Variants

**FIGURE 3:**
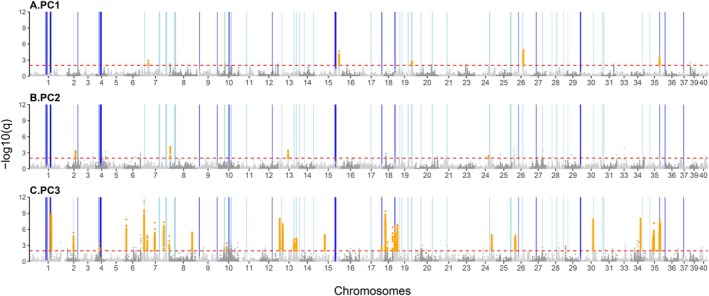
PCA‐based genome‐wide scans for putative regions under selection in Great Slave Lake's Lake Whitefish populations. Manhattan plots show genome‐wide patterns of divergence on PC1 (A), PC2 (B) and PC3 (C). Candidate outlier regions containing windows of 1000 SNPs with at least 10 outliers (*q* value < 0.01, red dashed line) are highlighted in orange. Clusters of over ten MDS outlier windows of 100 SNPs are highlighted in light blue. Regions identified visually as putative inversions are highlighted in dark blue. For visualization purposes, small chromosomes (22, 32, 38) with few SNPs were excluded.

Using a FastPCA selection scan in PCAngsd on the first 4 PC axes, we found a total of 6550 outlier SNPs (*q* < 0.01 (−log10(*q*) > 2); 616 outliers on PC1, 198 outliers on PC2, 5732 outliers on PC3 and 14 outliers on PC4. Only ten SNPs were outliers on more than one PC axis (PC1‐PC3: 7 SNPs, PC1‐PC2: 3 SNPs). Sixty‐three windows of 1000 SNPs contained at least 10 outliers. These windows were regrouped in thirty candidate regions (Figure 3; in orange; A) PC1: 5 regions, (B) PC2: 4 regions, (C) PC3: 21 regions; Table S2). No window contained at least 10 outliers on PC4. Only two of these thirty candidate regions, located on chromosomes 24 and 35, contained at least 10 outliers on multiple PC axes. On PC1, these regions of elevated genetic differentiation overlapped with 384 genes (Table S3.1), and gene ontology enrichment revealed five enriched biological processes, one enriched cellular component and seven enriched molecular functions (Table S4.1). Four enriched biological processes were related to immune functions, and one was related to eye development (Table S4.1). On PC2, regions of elevated genetic differentiation overlapped with 216 genes (Table S3.2), and gene ontology enrichment revealed eight enriched molecular functions, all related to ion transport channel activity (Table S4.2). On PC3, regions of elevated genetic differentiation overlapped with 2985 genes (Table S3.3), and gene ontology enrichment revealed 24 enriched biological processes, 10 enriched cellular components and 17 enriched molecular functions (Table S4.3). Most enriched biological processes were related to metabolic functions and immunity (Table S4.3).

Using a local PCA approach, we detected 62 regions, varying between 454.5 and 5221.7 Kbp in size, displaying patterns of genetic variation distinct from the genome‐wide signal on the first 5 MDS axes (Figure 3, in light blue; Table S5). These were regions that contained more than 10 outlier windows (> 3SD) of 100 SNPs distant by less than 20 windows. Out of these 62 regions, 20 were identified as putative inversions (Figure 3, in dark blue; Table S5), as they showed a three‐group pattern of the PC1 axis of a local PCA with elevated heterozygosity in the putative “AB” group.

**FIGURE 4:**
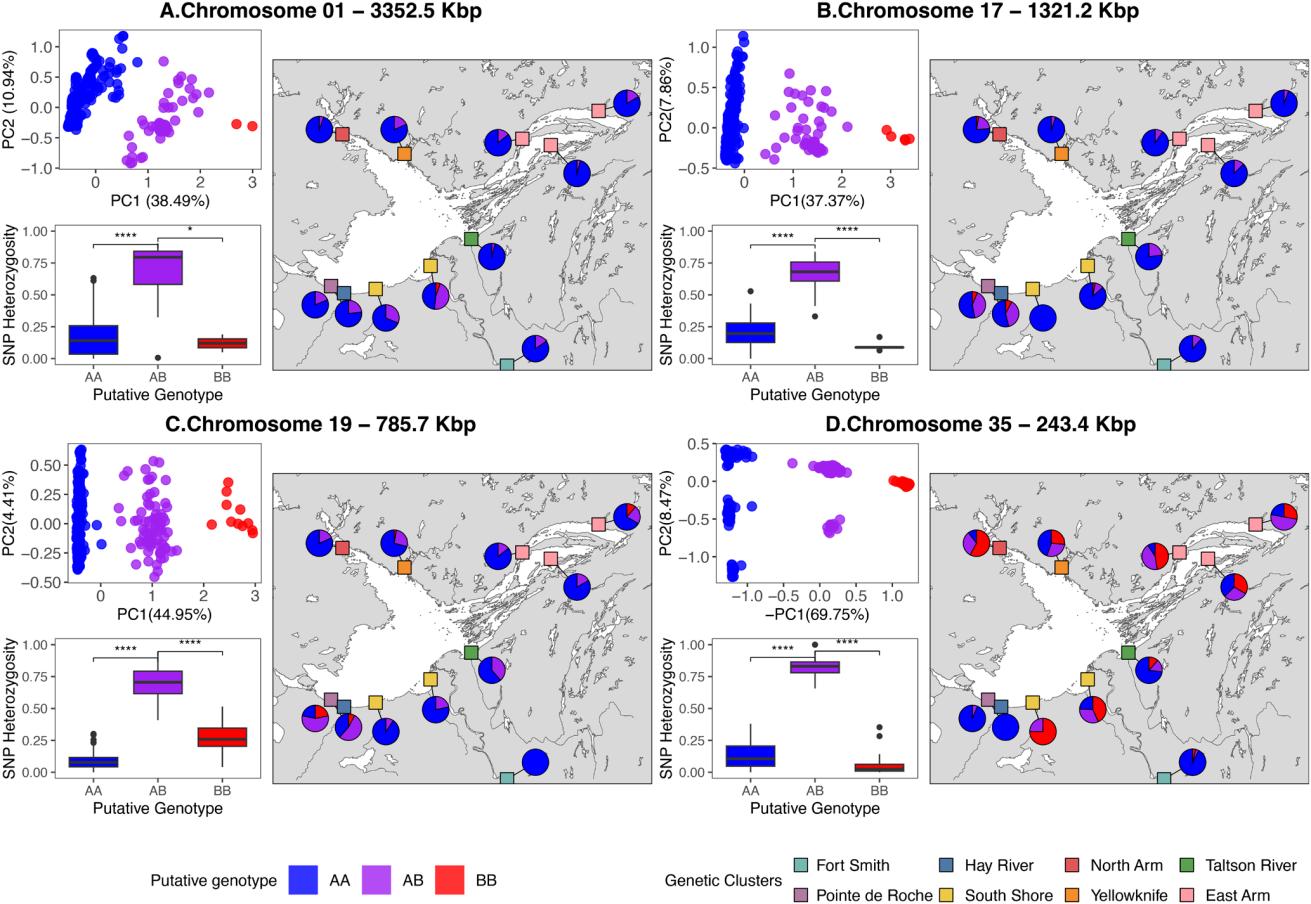
Putative inversions located on chromosomes 1 (A), 17 (B), 19 (C), and 35 (D). Top left: Local PCA of this region that suggests the presence of a chromosomal inversion, with each group on the PC1 representing a specific genotype. Bottom left: SNP heterozygosity in each of the three PC1 clusters, demonstrating that the “middle cluster” is the most heterozygous. The statistical significance of pairwise Wilcoxon tests are indicated with stars (****:*p* value ≤ 0.0001; *: *p* value ≤ 0.05). Right: Frequency of each genotype of the putative inversion at each sampling site, with the colours of the sampling site squares representing the dominant population at these locations. For visualization purposes, the Hay River genetic cluster (in blue) was moved to the mouth of Hay River, even though they were sampled at the same sampling site as the Pointe de Roche genetic cluster (in purple).

**FIGURE 5:**
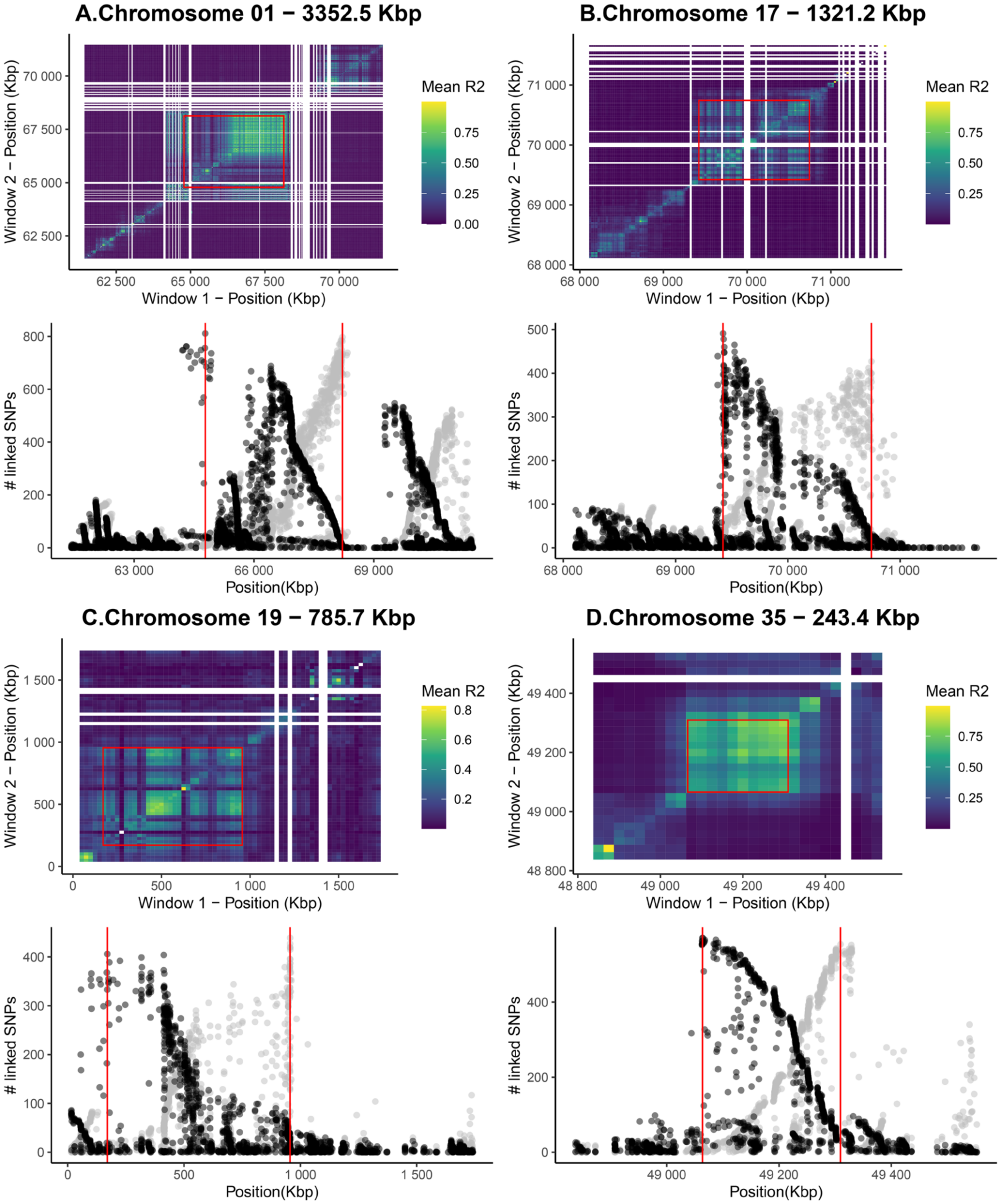
LD patterns in putative inversions located on chromosomes 1 (A), 17 (B), 19 (C) and 35 (D). Top: Heatmap showing mean *R* ^2^ values between 25 Kbp windows. Bottom: Number of highly linked (> top 5% *R* ^2^ of chromosome) upstream SNPs (grey points) and downstream SNPs (black points), highlighting LD breaks before and after putative inversion boundaries (red lines).

Fourteen MDS outlier regions overlapped with candidate regions found through the FastPCA selection scan. Four of these regions of elevated genetic differentiation overlapped with putative inversions. These putative inversions were located on chromosomes 1, 17, 19, and 35 (Figure 4). The frequency of the putative genotypes of each inversion varied between sampling sites (Figure 4). After investigating LD patterns in these putative inversions, we inferred that they were in LD blocks, as *R* ^2^ was significantly elevated in these regions compared to other blocks of the same size on the same chromosome (Table 2). After redefining inversion boundaries (Figure 5), the putative inversions located on chromosomes 1, 17, 19, and 35 were 3352.5 Kbp, 1321.2 Kbp, 785.7 Kbp, and 243.4 Kbp in length, respectively (Figure 4; Table 2).

**TABLE 2:** Characteristics of putative chromosomal inversions overlapping with regions of elevated genetic differentiation.

Chromosome	MDS axis	Start	End	Top 5% *R* ^2^ chromosome	Start—boundary	End—boundary	Length (Kbp)	Mean *R* ^2^		
								In region	In other regions of the same length	*p* value
Chr01	1	65,517,496	69,404,044	0.77	64,780,347	68,132,863	3352.5	0.31	0.07	2.12 × 10^−5^
Chr17	1	69,410,066	70,730,996	0.54	69,422,725	70,743,943	1321.2	0.24	0.09	6.91 × 10^−4^
Chr19	–1	101,640	2,237,474	0.66	170,278	955,945	785.7	0.35	0.13	3.60 × 10^−4^
Chr35	1	49,062,892	49,613,994	0.66	49,066,208	49,309,591	243.4	0.61	0.19	1.68 × 10^−5^

These four putative inversions overlapped with 151 genes (Table S3.4), with 67 on chromosome 1, 31 on chromosome 17, 42 on chromosome 19, and 10 on chromosome 35. Gene ontology analyses did not reveal any enriched biological or chemical processes associated with these inversions alone.

## 4 Discussion

Using low‐coverage whole‐genome resequencing, we inferred the presence of seven distinct genetic clusters at nine sampling locations in Great Slave Lake and one distinct genetic cluster in the Slave River. Genetic differentiation between sampling sites was consistent with an isolation‐by‐distance pattern. However, genetic structure varied among regions, with relatively higher fine‐scale genetic structure among sampling sites in environmentally heterogeneous sections of the Main Basin and comparatively weaker genetic differentiation despite large geographic distances in the environmentally homogeneous East Arm. This could suggest that local adaptation might play a role in shaping genetic structure in this large subarctic lake. Using genome‐wide scans, we detected multiple regions of elevated genetic differentiation, with some overlapping with putative structural variants displaying patterns consistent with the presence of chromosomal inversions. These results highlight the potential role of chromosomal rearrangements in the maintenance of genetic differentiation between putatively locally adapted populations in the face of gene flow in environmentally heterogeneous lakes.

### 4.1 Genetic Structure

Genetic differentiation between sampling sites in our system followed an isolation‐by‐distance (IBD) pattern, which is expected when gene flow is higher between geographically proximate locations (Wright 1943). This isolation‐by‐distance pattern was stronger between sites when the FOS site, located in the Slave River, was considered, which could suggest stronger constraints to dispersal in this river population compared to lake populations. This could also be due to the statistical properties of the Mantel Test, which can yield different results depending on the spatial scale used (Rousset 1997; Castric and Bernatchez 2003). In Great Slave Lake, capture‐recapture data suggest that Lake Whitefish may have relatively small spatial niches and might not often disperse to further locations, even if they have the capacity to do so (Keleher 1963). This behaviour, combined with putative spawning site fidelity observed in shoal‐spawning Lake Whitefish (Ebener et al. 2010, 2021; Ryther et al. 2024; Walker et al. 1993), could have led to reduced rates of long‐distance straying, resulting in the observed IBD pattern. Similar IBD patterns have been observed in Lake Whitefish from other large lakes (Shi et al. 2022). In Lake Michigan, for instance, Lake Whitefish display spatial segregation that results in temporally stable genetic structure (Nathan et al. 2016).

However, our data suggest that fine‐scale genetic structure in Great Slave Lake might have been shaped by factors other than IBD. For instance, in the productive, shallow and environmentally heterogeneous Western Basin of Great Slave Lake, multiple genetically distinct populations are maintained despite relatively short geographic distances. In this region, which encompasses the most heavily exploited fisheries management areas (FMAs IW and IE), three genetically distinct populations, Pointe de Roche, Hay River and South Shore, were identified at two sampling locations (PDR & BUR) distant by less than 60 km. Such genetic structure at small spatial scales can be shaped by the interaction of local adaptation and gene flow in heterogeneous environments, as effective dispersal can be reduced between locations with different selective regimes (Dionne et al. 2008; Hendry 2004; Nosil et al. 2005). In heterogeneous landscapes, adaptive divergence between populations could thus lead to increased spawning site fidelity, due to decreased fitness of strays (Hendry et al. 2003). In the Western Basin, spawning site fidelity could have been reinforced through spawning habitat differences, as Lake Whitefish in Great Slave Lake can either spawn in shallow nearshore habitats (Richardson et al. 2001) or use stream or river mouths (e.g., Hay River; Stewart 1999). It could also have been reinforced through divergent spawning timing (isolation‐by‐time; Hendry and Day 2005), as distinct Whitefish spawning aggregations have been observed at different times in FMA IW (Scott and Wheaton 1954).

Putative adaptive divergence in the Western Basin was further suggested by genome scans, which revealed multiple outlier regions on PC1 and PC3. These PC‐axes respectively discriminated the South Shore genetic cluster (BUR and FOR sites) and the Hay River and Pointe du Roche genetic clusters (PDR site) from the rest of the Great Slave Lake populations. On PC1, regions of elevated genetic differentiation, putatively under selection, overlapped with 13 genes, mostly γ‐crystallin‐like proteins, involved in eye lens development (Slingsby et al. 2013). In Great Slave Lake, differences in water clarity between regions could drive divergent selection on genes that provide better visual capabilities in turbid environments, such as those found near the Slave River outflows, which could serve as refuges from visual predators, especially for juveniles (Fiksen et al. 2002; Utne‐Palm 2002; reviewed in Zanghi and Ioannou 2025). Genes involved in visual capabilities have been thoroughly studied as a key adaptive trait in large lakes, notably in deep‐water fishes (e.g., Eaton et al. 2020; Van Nynatten et al. 2024). Moreover, γ‐crystallin‐like proteins might have retained some ancestral functions unrelated to vision (Weadick and Chang 2009) as they are homologous to some metabolic enzymes (e.g., lactate dehydrogenase; Jones et al. 1999). In *Coregonus clupeaformis* , they are expressed in muscles and have been hypothesized to be involved in metabolic processes related to swimming, especially in the dwarf ecotype that exploits the limnetic environment (Derome and Bernatchez 2006; Derome et al. 2006). On PC3, genome scans revealed that genetic differentiation was maintained in multiple outlier regions putatively under selection, with fourteen overlapping with low‐recombining regions and four displaying patterns consistent with the presence of chromosomal inversions. In heterogeneous environments, the interaction of linkage, divergent selection and gene flow can lead to increasingly concentrated genetic architecture (Nosil et al. 2009; Quilodrán et al. 2020; Yeaman 2013; Yeaman et al. 2016), with most genetic differentiation being maintained in low‐recombining regions that are protected from the homogenizing influence of gene flow (Akopyan et al. 2025; Samuk et al. 2017; Tigano and Friesen 2016). For instance, chromosomal inversions can often harbour co‐adapted large‐effect loci underlying important phenotypic traits (e.g., alternative migratory tactics; Arostegui et al. 2019; environmental adaptations; Matschiner et al. 2022; Stenløkk et al. 2022), as reduced levels of recombination between inverted and non‐inverted karyotypes shelter locally adapted alleles from gene flow (Kirkpatrick and Barton 2006). In the Western Basin, putative inversion allele frequencies on chromosomes 17, 19 and 35 varied greatly between sampling sites, which could further imply their role in shaping genetic divergence in this environmentally heterogeneous part of the lake. In large lakes, other studies have suggested that structural variation might play a role in maintaining local adaptation and genetic differentiation between populations (e.g., Thorstensen et al. 2022). For instance, in the Laurentian Great Lakes, a putative inversion on chromosome 20 was found to be involved in the genetic differentiation of Lake Whitefish populations in Lake Michigan (Shi et al. 2022) and Lake Erie (Euclide et al. 2022). However, it is also important to note that while the local PCA approach used in our analysis hints at the presence of chromosomal inversions, we cannot exclude other mechanisms that suppress recombination (see Ishigohoka et al. 2024). Furthermore, we cannot conclude that the regions of elevated genetic differentiation we detected arose through selective processes, as drift and other neutral processes can lead to similar patterns (e.g., Campagna et al. 2015; Dallaire et al. 2025; Quilodrán et al. 2020; Wang et al. 2016).

In other regions of Great Slave Lake, weaker genetic differentiation and elevated admixture suggested weaker barriers to gene flow among populations. For instance, populations from the Northern Basin were genetically similar to populations from both the Western/Central Basin and the East Arm. Indeed, Whitefish that spawned at the NOA site in a shallow section of the Northern Basin, were only weakly differentiated from individuals from the BUR and FOR sites, in the Western/Central Basin. This could be explained by the environmental similarity between the shallow section of the North Arm where the spawning site is located and some sections of the Main Basin, which harbour a similar ichtyofauna (Rawson 1951). Similarly, the weak genetic differentiation between the YEK site and the East Arm sites (TAN, LUK & MCB) could be, in part, explained by the environmental similarity between the deep Yellowknife Bay and the East Arm. In the Northern Basin, genetic discreteness among populations could also be influenced by their proximity to large rivers that potentially harbour adfluvial populations of Lake Whitefish. For instance, the Yellowknife River and the Taltson River have both been suggested to harbour fall spawning runs of Lake Whitefish (Baldwin et al. 2017; Bill et al. 1996; Miller et al. 2025). In the East Arm, the lack of genetic differentiation between the three relatively distant sampling locations (LUK, TAN & MCB) might suggest weaker spawning site fidelity in this region. Those patterns are consistent with the relative environmental homogeneity of the East Arm, which is less influenced by inflows from the Slave River (Evans 2000; Rawson 1950; Zhu et al. 2017). Lake Whitefish are also generally less abundant in the cold and deep waters of the East Arm (Keleher 1972; e.g., McLeod Bay, Rawson 1951). Northeastern populations have even been suggested to be reliant on immigration to sustain themselves (Kennedy 1953; but see Keleher 1963). As such, effective dispersal might be common in this region, resulting in weaker genetic differentiation among sites and more frequent admixture with nearby Northern Basin populations (NOA, YEK). The relatively elevated autosomal heterozygosity and genomic diversity (Θ_W_) of individuals from the TAN and MCB sites could be consistent with frequent gene flow from immigrants.

Finally, our results show that Lake Whitefish sampled at the FOS site, in the Slave River, the main tributary of the lake, were strongly genetically distinct from those sampled in Great Slave Lake. This is not surprising, as the Whitefish that spawn in the Rapids of the Drowned in Fort Smith have been suggested to be adfluvial, with adults migrating to Great Slave Lake in the summer to feed and returning to these large rapids to spawn during the fall (Baldwin et al. 2017; McLeod et al. 1985; Tallman 1996; Tallman et al. 2005; Tripp et al. 1981). However, while tagged fish have been shown to migrate to the lake, tagging data (McLeod et al. 1985; Stewart 1999; Tripp et al. 1981) and Traditional Ecological Knowledge (Bill et al. 1996) also suggest the presence of resident riverine whitefish in the Slave River. If the Fort Smith population is indeed adfluvial, gene flow with lake‐spawning fish could be reduced by philopatric behaviour, which is common in potamodromous migratory fish (Northcote 1997). Isolation‐by‐time (Hendry and Day 2005) could also reduce gene flow between putatively adfluvial and lake‐spawning populations, as migrating pre‐spawners such as those collected for this study start to appear at the base of the rapids in August (Tallman 1996), well before Lake Whitefish start spawning in Great Slave Lake (Richardson et al. 2001). Similar elevated genetic differentiation between river‐spawning and lake‐spawning Whitefish was observed in nearby Lake Athabasca (Gibelli et al. 2026).

### 4.2 Management Implications & Future Research Avenues

In Great Slave Lake, Lake Whitefish populations have been managed through annual commercial quotas in six of the seven fisheries management areas (FMAs IW, IE, II, III, IV and V), with FMA VI being closed to commercial fishing (Department of Fisheries and Oceans [DFO] 2015). Our results support that these management areas do not accurately reflect the complexity of Lake Whitefish stocks, which are composed of at least eight genetically distinct populations. For instance, in the Western Basin (FMAs IW, IE), which supports most of the commercial activities, three populations probably overlap during the summer, with some contributing more to mixed‐stock fisheries than others. Akin to what is observed in the Laurentian Great Lakes (Ebener et al. 2010, 2021), distinct populations might differ in their spatial use, with some having larger distributions than others. Our results suggest, for instance, that the Pointe du Roche population, which was exclusively found at the PDR site, might have a relatively small spatial distribution, as no fish from this population were caught at the nearby BUR site. Future acoustic telemetry work combined with genetic stock identification and mixed‐stock analysis should shed light on the distribution of these populations across multiple fisheries management areas boundaries in the summer. As such, the eight populations delineated in the current study will serve as a baseline for future mixed‐stock analysis and genetic stock identification of commercial landings using a recently developed GT‐seq panel (Beemelmanns et al. 2025). The potential importance of potamodromous populations in this system and their contribution to commercial fisheries should also be more thoroughly investigated. For instance, future work on unsampled adfluvial populations that migrate in other rivers, like the Little Buffalo River (Roberge et al. 1985), could reveal genetically distinct populations.

During the fall spawning season, closure zones that shelter shallow nearshore areas from commercial activities could protect certain populations. As such, the South Shore population, which spawns at the BUR and FOR sites, is potentially protected from commercial harvesting during the spawning season, as those sites are located in closure zones. Whitefish from this population were also found exclusively at these two sites. Similarly, the NOA, TAR, and YEK sites, which each support distinct populations, are located directly in closure zones. However, FMA IW supports at least two spawning populations (Pointe du Roche, Hay River) that could be left unprotected, as no closure zones protect spawning habitats west of Pointe de Roche (PDR) in the fall.

While our study supports the genetic distinctness of some Lake Whitefish populations in Great Slave Lake, further investigation may be necessary to determine whether these populations have distinct phenotypic or life history traits. In Great Slave Lake, multiple putative morphotypes of Whitefish have been informally described (Kennedy 1953; Scott and Wheaton 1954; Rawson 1947), but have not been previously considered distinct designatable units (Mee et al. 2015; Rogers 2008). Indeed, since Great Slave Lake has only been colonized by the Mississippian glacial lineage (Bernatchez and Dodson 1991; Foote et al. 1992; Franzin and Clayton 1977), these putative morphotypes differ from the traditional benthic and limnetic species pair usually associated with secondary contact of different glacial lineages (Bernatchez and Dodson 1990). However, ecological niche differences could have driven sympatric morphological divergence in Lake Whitefish in this large lake, as documented for Lake Trout (Zimmerman et al. 2006; Hansen et al. 2016) and ciscoes (Muir et al. 2014).

### 4.3 Conclusion

Overall, this study is the first assessment of Lake Whitefish genetic structure in Great Slave Lake, which brings insight into the genetic connectivity of populations with important potential implications for the management of commercial stocks in this northern Great Lake. Our results echo those of Shi et al. (2022), which found stronger genetic structure among Lake Whitefish populations inhabiting the environmentally heterogeneous eastern side of Lake Michigan compared to the northwestern side. Taken together, these studies showcase the importance of environmental factors in shaping fine‐scale genetic structure in large, recently deglaciated lakes. Furthermore, our study highlights the utility of whole‐genome data for delineating shallow genetic structure and for studying genetic differentiation in contexts of high gene flow.

## Funding

This work was supported by Genome Canada, Génome Québec, Ontario Genomics, Fisheries and Oceans Canada, Ressources Aquatiques Québec, Natural Sciences and Engineering Research Council of Canada and Fonds de recherche du Québec – Nature et technologies.

## Conflicts of Interest

The authors declare no conflicts of interest.

## Supporting information


**Figure S1:** Mean individual depth of coverage per sample at each sampling location. Individuals from the Hay River genetic clusters are grouped separately.
**Figure S2.1:** Admixture evaluation as correlation of residuals in evalAdmix for K2 to K10.
**Figure S2.2:** Admixture evaluation as correlation of residuals in evalAdmix for K11 to K15.
**Figure S3.1:** Individual admixture proportions from *K* = 2 to *K* = 8. Each graph is the average iteration of the most supported mode according to *Clumppling*, based on the aggregation of 50 runs for each *K*.
**Figure S3.2:** Individual admixture proportions from *K* = 9 to *K* = 15. Each graph is the average iteration of the most supported mode according to *Clumppling*, based on the aggregation of 50 runs for each *K*.
**Figure S4:** Scree‐plot of the % of variance explained for each of the first 20 PC axes based on a matrix of covariance computed using 181,603 SNPs.

**Table S1.1:** Best number of *K* clusters according to Puechmaille estimators using different thresholds.
**Table S1.2:** Clumppling alignement statistics per K.
**Table S1.3:** Proportion of admixed Individuals at each sampling site at K = 8.
**Table S2:** Candidate regions composed of multiple windows of 1000 SNPs containing at least 10 FastPCA outliers on the first 3PC axes.
**Table S3.1:** Genes overlapping with regions of elevated genetic differentiation on PC1.
**Table S3.2:** Genes overlapping with regions of elevated genetic differentiation on PC2.
**Table S3.3:** Genes overlapping with regions of elevated genetic differentiation on PC3.
**Table S3.4:** Genes overlapping with regions of elevated genetic differentiation overlapping with putative chromosomal inversions.
**Table S4.1:** Enriched gene ontology terms for genes overlapping with regions of elevated genetic differentiation on PC1.
**Table S4.2:** Enriched gene ontology terms for genes overlapping with regions of elevated genetic differentiation on PC2.
**Table S4.3:** Enriched gene ontology terms for genes overlapping with regions of elevated genetic differentiation on PC3.
**Table S5:** Candidate outlier regions on 5 MDS axes according to a local PCA analysis.

## Data Availability

All raw sequencing data for this project were already deposited on the NCBI Short Read Archive (SRA) as part of another project (accession number: PRJNA1051576).

## References

[eva70268-bib-0001] Akopyan, M. , A. Tigano , A. Jacobs , A. P. Wilder , and N. O. Therkildsen . 2025. “Genetic Differentiation Is Constrained to Chromosomal Inversions and Putative Centromeres in Locally Adapted Populations With Higher Gene Flow.” Molecular Biology and Evolution 42, no. 5: msaf092. 10.1093/molbev/msaf092. 40247662 PMC12046131

[eva70268-bib-0002] Allendorf, F. W. , P. R. England , G. Luikart , P. A. Ritchie , and N. Ryman . 2008. “Genetic Effects of Harvest on Wild Animal Populations.” Trends in Ecology & Evolution 23, no. 6: 327–337. 10.1016/j.tree.2008.02.008. 18439706

[eva70268-bib-0003] Allendorf, F. W. , P. A. Hohenlohe , and G. Luikart . 2010. “Genomics and the Future of Conservation Genetics.” Nature Reviews Genetics 11, no. 10: 697–709. 10.1038/nrg2844. 20847747

[eva70268-bib-0004] Andersson, L. , D. Bekkevold , F. Berg , et al. 2024. “How Fish Population Genomics Can Promote Sustainable Fisheries: A Road Map.” Annual Review of Animal Biosciences 12: 1–20. 10.1146/annurev-animal-021122-102933. 37906837

[eva70268-bib-0005] Arostegui, M. C. , T. P. Quinn , L. W. Seeb , J. E. Seeb , and G. J. McKinney . 2019. “Retention of a Chromosomal Inversion From an Anadromous Ancestor Provides the Genetic Basis for Alternative Freshwater Ecotypes in Rainbow Trout.” Molecular Ecology 28, no. 6: 1412–1427. 10.1111/mec.15037. 30714254

[eva70268-bib-0006] Baldwin, C. , L. Bradford , M. Carr , et al. 2017. “Ecological Patterns of Fish Distribution in the Slave River Delta Region, Northwest Territories, Canada, as Relayed by Traditional Knowledge and Western Science.” International Journal of Water Resources Development 34, no. 2: 305–324. 10.1080/07900627.2017.1298516.

[eva70268-bib-0007] Baym, M. , S. Kryazhimskiy , T. D. Lieberman , H. Chung , M. M. Desai , and R. Kishony . 2015. “Inexpensive Multiplexed Library Preparation for Megabase‐Sized Genomes.” PLoS One 10, no. 5: e0128036. 10.1371/journal.pone.0128036. 26000737 PMC4441430

[eva70268-bib-0008] Beech, S. J. H. , C. W. Elliott , M. S. Ridgway , E. Brown , and B. L. Tufts . 2024. “Spatial Comparison of Two Lake Whitefish ( *Coregonus clupeaformis* ) Spawning Aggregations From the Bay of Quinte and Eastern Lake Ontario.” Journal of Great Lakes Research 50, no. 6: 102443. 10.1016/j.jglr.2024.102443.

[eva70268-bib-0009] Beemelmanns, A. , R. Bouchard , S. Michaelides , et al. 2025. “Development of SNP Panels From Low‐Coverage Whole Genome Sequencing (lcWGS) to Support Indigenous Fisheries for Three Salmonid Species in Northern Canada.” Molecular Ecology Resources 25, no. 3: e14040. 10.1111/1755-0998.14040. 39552382 PMC11887602

[eva70268-bib-0010] Benestan, L. 2020. “Population Genomics Applied to Fishery Management and Conservation.” In Population Genomics: Marine Organisms, edited by M. F. Oleksiak and O. P. Rajora , 399–421. Springer International Publishing. 10.1007/13836_2019_66.

[eva70268-bib-0011] Benjamini, Y. , and Y. Hochberg . 1995. “Controlling the False Discovery Rate: A Practical and Powerful Approach to Multiple Testing.” Journal of the Royal Statistical Society. Series B, Statistical Methodology 57, no. 1: 289–300. 10.1111/j.2517-6161.1995.tb02031.x.

[eva70268-bib-0012] Berdan, E. L. , N. H. Barton , R. Butlin , et al. 2023. “How Chromosomal Inversions Reorient the Evolutionary Process.” Journal of Evolutionary Biology 36, no. 12: 1761–1782. 10.1111/jeb.14242. 37942504

[eva70268-bib-0013] Bernatchez, L. , and J. J. Dodson . 1990. “Allopatric Origin of Sympatric Populations of Lake Whitefish ( *Coregonus clupeaformis* ) as Revealed by Mitochondrial‐DNA Restriction Analysis.” Evolution 44, no. 5: 1263–1271. 10.1111/j.1558-5646.1990.tb05230.x. 28563883

[eva70268-bib-0014] Bernatchez, L. , and J. J. Dodson . 1991. “Phylogeographic Structure in Mitochondrial DNA of the Lake Whitefish ( *Coregonus clupeaformis* ) and Its Relation to Pleistocene Glaciations.” Evolution 45, no. 4: 1016–1035. 10.1111/j.1558-5646.1991.tb04367.x. 28564052

[eva70268-bib-0015] Bernatchez, L. , M. Wellenreuther , C. Araneda , et al. 2017. “Harnessing the Power of Genomics to Secure the Future of Seafood.” Trends in Ecology & Evolution 32, no. 9: 665–680. 10.1016/j.tree.2017.06.010. 28818341

[eva70268-bib-0016] Bernatchez, L. , and C. C. Wilson . 1998. “Comparative Phylogeography of Nearctic and Palearctic Fishes.” Molecular Ecology 7, no. 4: 431–452. 10.1046/j.1365-294x.1998.00319.x.

[eva70268-bib-0017] Berner, D. 2019. “Allele Frequency Difference AFD–an Intuitive Alternative to FST for Quantifying Genetic Population Differentiation.” Genes 10, no. 4: 308. 10.3390/genes10040308. 31003563 PMC6523497

[eva70268-bib-0018] Bernos, T. A. , J. Gibelli , S. Michaelides , et al. 2024. “Widespread Admixture Blurs Population Structure and Confounds Lake Trout ( *Salvelinus namaycush* ) Conservation Even in the Genomic Era.” Scientific Reports 14, no. 1: 30838. 10.1038/s41598-024-81531-7. 39730611 PMC11680572

[eva70268-bib-0019] Bhatia, G. , N. Patterson , S. Sankararaman , and A. L. Price . 2013. “Estimating and Interpreting FST: The Impact of Rare Variants.” Genome Research 23, no. 9: 1514–1521. 10.1101/gr.154831.113. 23861382 PMC3759727

[eva70268-bib-0020] Bill, L. , J. Crozier , and D. C. Surrendi . 1996. A Report of Wisdom Synthesized From the Traditional Knowledge Component Studies (Northern River Basins Study Synthesis Report No. 12). Northern River Basins Study. https://publications.gc.ca/site/eng/60987/publication.html.

[eva70268-bib-0021] Bootsma, M. L. , K. M. Gruenthal , G. J. McKinney , et al. 2020. “A GT‐Seq Panel for Walleye ( *Sander vitreus* ) Provides Important Insights for Efficient Development and Implementation of Amplicon Panels in Non‐Model Organisms.” Molecular Ecology Resources 20, no. 6: 1706–1722. 10.1111/1755-0998.13226. 32668508

[eva70268-bib-0022] Bouchard, R. , C. Babin , E. Normandeau , et al. 2025. “Shared Dispersal Patterns but Contrasting Levels of Gene Flow in Two Anadromous Salmonids Along a Broad Subarctic Coastal Gradient.” Molecular Ecology 34, no. 9: e17739. 10.1111/mec.17739. 40108992 PMC12010461

[eva70268-bib-0023] Bradbury, I. R. , S. Hubert , B. Higgins , et al. 2013. “Genomic Islands of Divergence and Their Consequences for the Resolution of Spatial Structure in an Exploited Marine Fish.” Evolutionary Applications 6, no. 3: 450–461. 10.1111/eva.12026. 23745137 PMC3673473

[eva70268-bib-0024] Breese, M. R. , and Y. Liu . 2013. “NGSUtils: A Software Suite for Analyzing and Manipulating Next‐Generation Sequencing Datasets.” Bioinformatics 29, no. 4: 494–496. 10.1093/bioinformatics/bts731. 23314324 PMC3570212

[eva70268-bib-0025] Brenden, T. O. , R. W. Brown , M. P. Ebener , K. Reid , and T. J. Newcomb . 2012. “Great Lakes Commercial Fisheries: Historical Overview and Prognoses for the Future.” In Great Lakes Fisheries Policy and Management, 339–398. Michigan State University Press. 10.14321/j.ctt7ztc19.15.

[eva70268-bib-0026] Browning, B. L. 2013. BEAGLE Utilities [Computer Software]. https://faculty.washington.edu/browning/beagle_utilities/utilities.html.

[eva70268-bib-0027] Cabanettes, F. , and C. Klopp . 2018. “D‐GENIES: Dot Plot Large Genomes in an Interactive, Efficient and Simple Way.” PeerJ 6: e4958. 10.7717/peerj.4958. 29888139 PMC5991294

[eva70268-bib-0028] Camacho, C. , G. Coulouris , V. Avagyan , et al. 2009. “BLAST+: Architecture and Applications.” BMC Bioinformatics 10: 421. 10.1186/1471-2105-10-421. 20003500 PMC2803857

[eva70268-bib-0029] Campagna, L. , I. Gronau , L. Silveira , A. Siepel , and I. Lovette . 2015. “Distinguishing Noise From Signal in Patterns of Genomic Divergence in a Highly Polymorphic Avian Radiation.” Molecular Ecology 24, no. 16: 4238–4251. 10.1111/mec.13314. 26175196

[eva70268-bib-0030] Castric, V. , and L. Bernatchez . 2003. “The Rise and Fall of Isolation by Distance in the Anadromous Brook Charr ( *Salvelinus fontinalis* Mitchill).” Genetics 163, no. 3: 983–996. 10.1093/genetics/163.3.983. 12663537 PMC1462472

[eva70268-bib-0031] Cayuela, H. , Q. Rougemont , M. Laporte , et al. 2020. “Shared Ancestral Polymorphisms and Chromosomal Rearrangements as Potential Drivers of Local Adaptation in a Marine Fish.” Molecular Ecology 29, no. 13: 2379–2398. 10.1111/mec.15499. 32497342

[eva70268-bib-0032] Chen, S. , Y. Zhou , Y. Chen , and J. Gu . 2018. “Fastp: An Ultra‐Fast All‐In‐One FASTQ Preprocessor.” Bioinformatics 34, no. 17: i884–i890. 10.1093/bioinformatics/bty560. 30423086 PMC6129281

[eva70268-bib-0033] Dallaire, X. , R. Bouchard , P. Hénault , et al. 2023. “Widespread Deviant Patterns of Heterozygosity in Whole‐Genome Sequencing due to Autopolyploidy, Repeated Elements, and Duplication.” Genome Biology and Evolution 15, no. 12: evad229. 10.1093/gbe/evad229. 38085037 PMC10752349

[eva70268-bib-0034] Dallaire, X. , E. Normandeau , T. Brazier , et al. 2025. “Leveraging Whole Genomes, Mitochondrial DNA and Haploblocks to Decipher Complex Demographic Histories: An Example From a Broadly Admixed Arctic Fish.” Molecular Ecology 34, no. 10: e17772. 10.1111/mec.17772. 40289656 PMC12051761

[eva70268-bib-0035] Danecek, P. , J. K. Bonfield , J. Liddle , et al. 2021. “Twelve Years of SAMtools and BCFtools.” GigaScience 10, no. 2: giab008. 10.1093/gigascience/giab008. 33590861 PMC7931819

[eva70268-bib-0036] Delgado, M. L. , M. Van Wyngaarden , A. L. Einfeldt , et al. 2025. “The Genomic Consequences of Fisheries Collapse in a Marine Fish.” ICES Journal of Marine Science 82, no. 9: fsaf155. 10.1093/icesjms/fsaf155.

[eva70268-bib-0037] Department of Fisheries and Oceans [DFO] . 2015. *Assessment of Lake Whitefish Status in Great Slave Lake, Northwest Territories, Canada, 1972–2004*. (DFO Canadian Science Advisory Secretariat (CSAS) 2015/042; Science Advisory Report). https://waves‐vagues.dfo‐mpo.gc.ca/library‐bibliotheque/365071.pdf.

[eva70268-bib-0038] Derome, N. , and L. Bernatchez . 2006. “The Transcriptomics of Ecological Convergence Between 2 Limnetic Coregonine Fishes (Salmonidae).” Molecular Biology and Evolution 23, no. 12: 2370–2378. 10.1093/molbev/msl110. 16963516

[eva70268-bib-0039] Derome, N. , P. Duchesne , and L. Bernatchez . 2006. “Parallelism in Gene Transcription Among Sympatric Lake Whitefish ( *Coregonus clupeaformis* Mitchill) Ecotypes.” Molecular Ecology 15, no. 5: 1239–1249. 10.1111/j.1365-294X.2005.02968.x. 16626451

[eva70268-bib-0040] Des Roches, S. , L. H. Pendleton , B. Shapiro , and E. P. Palkovacs . 2021. “Conserving Intraspecific Variation for Nature's Contributions to People.” Nature Ecology & Evolution 5, no. 5: 574–582. 10.1038/s41559-021-01403-5. 33649544

[eva70268-bib-0041] Dionne, M. , F. Caron , J. J. Dodson , and L. Bernatchez . 2008. “Landscape Genetics and Hierarchical Genetic Structure in Atlantic Salmon: The Interaction of Gene Flow and Local Adaptation.” Molecular Ecology 17, no. 10: 2382–2396. 10.1111/j.1365-294X.2008.03771.x. 18430145

[eva70268-bib-0042] DuFour, M. R. , C. J. May , E. F. Roseman , et al. 2015. “Portfolio Theory as a Management Tool to Guide Conservation and Restoration of Multi‐Stock Fish Populations.” Ecosphere 6, no. 12: 1–21. 10.1890/ES15-00237.1.

[eva70268-bib-0043] Dupont, P.‐P. , V. Bourret , and L. Bernatchez . 2007. “Interplay Between Ecological, Behavioural and Historical Factors in Shaping the Genetic Structure of Sympatric Walleye Populations ( *Sander vitreus* ).” Molecular Ecology 16, no. 5: 937–951. 10.1111/j.1365-294X.2006.03205.x. 17305852

[eva70268-bib-0044] Eaton, K. M. , M. A. Bernal , N. J. C. Backenstose , D. L. Yule , and T. J. Krabbenhoft . 2020. “Nanopore Amplicon Sequencing Reveals Molecular Convergence and Local Adaptation of Rhodopsin in Great Lakes Salmonids.” Genome Biology and Evolution 13, no. 2: evaa237. 10.1093/gbe/evaa237. PMC7874997 33247716

[eva70268-bib-0045] Ebener, M. , T. Brenden , G. Wright , M. Jones , and M. Faisal . 2010. “Spatial and Temporal Distributions of Lake Whitefish Spawning Stocks in Northern Lakes Michigan and Huron, 2003–2008.” Journal of Great Lakes Research 36, no. 1: 38–51. 10.1016/j.jglr.2010.02.002.

[eva70268-bib-0046] Ebener, M. P. , E. S. Dunlop , and A. M. Muir . 2021. Declining Recruitment of Lake Whitefish to Fisheries in the Laurentian Great Lakes: Management Considerations and Research Priorities [Miscellaneous Publication]. Great Lakes Fishery Commission. https://www.glfc.org/pubs/misc/2021‐01.pdf.

[eva70268-bib-0047] Elmer, K. R. , J. K. J. Van Houdt , A. Meyer , and F. A. M. Volckaert . 2008. “Population Genetic Structure of North American Burbot ( *Lota lota* Maculosa) Across the Nearctic and at Its Contact Zone With Eurasian Burbot ( *Lota lota* Lota).” Canadian Journal of Fisheries and Aquatic Sciences 65, no. 11: 2412–2426. 10.1139/F08-173.

[eva70268-bib-0048] Euclide, P. T. , H. Kuhl , C. C. Wilson , et al. 2024. “Human Impacts on Great Lakes Walleye *Sander vitreus* Structure, Diversity and Local Adaptation.” Molecular Ecology 34: e17558. 10.1111/mec.17558. 39487667 PMC12684333

[eva70268-bib-0049] Euclide, P. T. , W. A. Larson , Y. Shi , et al. 2024. “Conserved Islands of Divergence Associated With Adaptive Variation in Sockeye Salmon Are Maintained by Multiple Mechanisms.” Molecular Ecology 33, no. 24: e17126. 10.1111/mec.17126. 37695544 PMC11628665

[eva70268-bib-0050] Euclide, P. T. , J. D. Schmitt , R. T. Kraus , A. Cook , and J. Markham . 2022. “Genome‐Wide Genetic Diversity May Help Identify Fine‐Scale Genetic Structure Among Lake Whitefish Spawning Groups in Lake Erie.” Journal of Great Lakes Research 48, no. 5: 1298–1305. 10.1016/j.jglr.2022.05.020.

[eva70268-bib-0051] Evans, M. S. 2000. “The Large Lake Ecosystems of Northern Canada.” Aquatic Ecosystem Health & Management 3, no. 1: 65–79. 10.1080/14634980008656992.

[eva70268-bib-0052] Fee, E. J. , M. P. Stainton , and H. J. Kling . 1985. Primary Production and Related Limnological Data for Some Lakes of the Yellowknife, NWT Area (Canadian Technical Report of Fisheries and Aquatic Sciences 1409; p. v + 55p.). Department of Fisheries. https://publications.gc.ca/collections/collection_2013/mpo‐dfo/Fs97‐6‐1409‐eng.pdf.

[eva70268-bib-0053] Fiksen, Ø. , D. L. Aksnes , M. H. Flyum , and J. Giske . 2002. “The Influence of Turbidity on Growth and Survival of Fish Larvae: A Numerical Analysis.” Hydrobiologia 484, no. 1: 49–59. 10.1023/A:1021396719733.

[eva70268-bib-0054] Foote, C. J. , J. W. Clayton , C. C. Lindsey , and R. A. Bodaly . 1992. “Evolution of Lake Whitefish ( *Coregonus clupeaformis* ) in North America During the Pleistocene: Evidence for a Nahanni Glacial Refuge Race in the Northern Cordillera Region.” Canadian Journal of Fisheries and Aquatic Sciences 49, no. 4: 760–768. 10.1139/f92-085.

[eva70268-bib-0055] Forester, B. R. , M. Murphy , C. Mellison , et al. 2022. “Genomics‐Informed Delineation of Conservation Units in a Desert Amphibian.” Molecular Ecology 31, no. 20: 5249–5269. 10.1111/mec.16660. 35976166 PMC9804278

[eva70268-bib-0056] Fox, E. A. , A. E. Wright , M. Fumagalli , and F. G. Vieira . 2019. “ngsLD: Evaluating Linkage Disequilibrium Using Genotype Likelihoods.” Bioinformatics 35, no. 19: 3855–3856. 10.1093/bioinformatics/btz200. 30903149

[eva70268-bib-0057] Franzin, W. G. , and J. W. Clayton . 1977. “A Biochemical Genetic Study of Zoogeography of Lake Whitefish ( *Coregonus clupeaformis* ) in Western Canada.” Journal of the Fisheries Research Board of Canada 34, no. 5: 617–625. 10.1139/f77-097.

[eva70268-bib-0058] Fraser, D. J. , and L. Bernatchez . 2005. “Adaptive Migratory Divergence Among Sympatric Brook Charr Populations.” Evolution; International Journal of Organic Evolution 59, no. 3: 611–624. 10.1554/04-346. 15856703

[eva70268-bib-0059] Fuentes‐Pardo, A. P. , and D. E. Ruzzante . 2017. “Whole‐Genome Sequencing Approaches for Conservation Biology: Advantages, Limitations and Practical Recommendations.” Molecular Ecology 26, no. 20: 5369–5406. 10.1111/mec.14264. 28746784

[eva70268-bib-0060] Funk, W. C. , J. K. McKay , P. A. Hohenlohe , and F. W. Allendorf . 2012. “Harnessing Genomics for Delineating Conservation Units.” Trends in Ecology & Evolution 27, no. 9: 489–496. 10.1016/j.tree.2012.05.012. 22727017 PMC4185076

[eva70268-bib-0061] Gagnaire, P.‐A. , T. Broquet , D. Aurelle , et al. 2015. “Using Neutral, Selected, and Hitchhiker Loci to Assess Connectivity of Marine Populations in the Genomic Era.” Evolutionary Applications 8, no. 8: 769–786. 10.1111/eva.12288. 26366195 PMC4561567

[eva70268-bib-0062] Galinsky, K. J. , G. Bhatia , P.‐R. Loh , et al. 2016. “Fast Principal‐Component Analysis Reveals Convergent Evolution of ADH1B in Europe and East Asia.” American Journal of Human Genetics 98, no. 3: 456–472. 10.1016/j.ajhg.2015.12.022. 26924531 PMC4827102

[eva70268-bib-0063] Garcia‐Erill, G. , and A. Albrechtsen . 2020. “Evaluation of Model Fit of Inferred Admixture Proportions.” Molecular Ecology Resources 20, no. 4: 936–949. 10.1111/1755-0998.13171. 32323416

[eva70268-bib-0064] Gibelli, J. , S. Michaelides , H. Won , et al. 2026. “Intraspecific Complexity in Mercury Contamination of Two Harvested Fishes Revealed by Genetics: Food Security and Conservation Implications.” Science of the Total Environment 1011: 181133. 10.1016/j.scitotenv.2025.181133. 41380599

[eva70268-bib-0065] Hansen, M. J. , N. A. Nate , L. Chavarie , A. M. Muir , M. S. Zimmerman , and C. C. Krueger . 2016. “Life History Differences Between Fat and Lean Morphs of Lake Charr ( *Salvelinus namaycush* ) in Great Slave Lake, Northwest Territories, Canada.” Hydrobiologia 783, no. 1: 21–35. 10.1007/s10750-015-2633-2.

[eva70268-bib-0066] Hendry, A. P. 2004. “Selection Against Migrants Contributes to the Rapid Evolution of Ecologically Dependent Reproductive Isolation.” Evolutionary Ecology Research 6: 1219–1226. https://www.evolutionary‐ecology.com/issues/v06n08/jjar1742.pdf.

[eva70268-bib-0067] Hendry, A. P. , V. Castric , M. T. Kinnison , and T. P. Quinn . 2003. “The Evolution of Philopatry and Dispersal—Homing Versus Straying in Salmonids.” In Evolution Illuminated, edited by A. P. Hendry and S. C. Stearns , 52–91. Oxford University Press. 10.1093/oso/9780195143850.003.0003.

[eva70268-bib-0068] Hendry, A. P. , and T. Day . 2005. “Population Structure Attributable to Reproductive Time: Isolation by Time and Adaptation by Time.” Molecular Ecology 14, no. 4: 901–916. 10.1111/j.1365-294X.2005.02480.x. 15773924

[eva70268-bib-0069] Hilborn, R. , T. P. Quinn , D. E. Schindler , and D. E. Rogers . 2003. “Biocomplexity and Fisheries Sustainability.” Proceedings of the National Academy of Sciences of the United States of America 100, no. 11: 6564–6568. 10.1073/pnas.1037274100. 12743372 PMC164486

[eva70268-bib-0070] Hoffmann, A. A. , and L. H. Rieseberg . 2008. “Revisiting the Impact of Inversions in Evolution: From Population Genetic Markers to Drivers of Adaptive Shifts and Speciation?” Annual Review of Ecology, Evolution, and Systematics 39: 21–42. 10.1146/annurev.ecolsys.39.110707.173532. PMC2858385 20419035

[eva70268-bib-0071] Hohenlohe, P. A. , W. C. Funk , and O. P. Rajora . 2021. “Population Genomics for Wildlife Conservation and Management.” Molecular Ecology 30, no. 1: 62–82. 10.1111/mec.15720. 33145846 PMC7894518

[eva70268-bib-0072] Homola, J. J. , K. T. Scribner , E. A. Baker , and N. A. Auer . 2010. “Genetic Assessment of Straying Rates of Wild and Hatchery Reared Lake Sturgeon ( *Acipenser fulvescens* ) in Lake Superior Tributaries.” Journal of Great Lakes Research 36, no. 4: 798–802. 10.1016/j.jglr.2010.08.011.

[eva70268-bib-0073] Homola, J. J. , K. T. Scribner , R. F. Elliott , et al. 2012. “Genetically Derived Estimates of Contemporary Natural Straying Rates and Historical Gene Flow Among Lake Michigan Lake Sturgeon Populations.” Transactions of the American Fisheries Society 141, no. 5: 1374–1388. 10.1080/00028487.2012.694829.

[eva70268-bib-0075] Huang, K. , R. L. Andrew , G. L. Owens , K. L. Ostevik , and L. H. Rieseberg . 2020. “Multiple Chromosomal Inversions Contribute to Adaptive Divergence of a Dune Sunflower Ecotype.” Molecular Ecology 29, no. 14: 2535–2549. 10.1111/mec.15428. 32246540

[eva70268-bib-0076] Ishigohoka, J. , K. Bascón‐Cardozo , A. Bours , et al. 2024. “Distinct Patterns of Genetic Variation at Low‐Recombining Genomic Regions Represent Haplotype Structure.” Evolution 78, no. 12: 1916–1935. 10.1093/evolut/qpae117. 39208288

[eva70268-bib-0077] Jenkins, T. L. 2024. “Mapmixture: An R Package and Web App for Spatial Visualisation of Admixture and Population Structure.” Molecular Ecology Resources 24, no. 4: e13943. 10.1111/1755-0998.13943. 38390660

[eva70268-bib-0078] Jones, S. E. , C. Jomary , J. Grist , J. Makwana , and M. J. Neal . 1999. “Retinal Expression of γ‐Crystallins in the Mouse.” Investigative Ophthalmology & Visual Science 40, no. 12: 3017–3020. 10549666

[eva70268-bib-0079] Kawecki, T. J. , and D. Ebert . 2004. “Conceptual Issues in Local Adaptation.” Ecology Letters 7, no. 12: 1225–1241. 10.1111/j.1461-0248.2004.00684.x.

[eva70268-bib-0080] Keleher, J. J. 1963. “The Movement of Tagged Great Slave Lake Fish.” Journal of the Fisheries Research Board of Canada 20, no. 2: 319–326. 10.1139/f63-028.

[eva70268-bib-0081] Keleher, J. J. 1972. “Great Slave Lake: Effects of Exploitation on the Salmonid Community.” Journal of the Fisheries Research Board of Canada 29, no. 6: 741–753. 10.1139/f72-119.

[eva70268-bib-0082] Kennedy, W. A. 1953. “Growth, Maturity, Fecundity and Mortality in the Relatively Unexploited Whitefish, *Coregonus clupeaformis* , of Great Slave Lake.” Journal of the Fisheries Research Board of Canada 10, no. 7: 413–441. 10.1139/f53-025.

[eva70268-bib-0083] Kirkpatrick, M. , and N. Barton . 2006. “Chromosome Inversions, Local Adaptation and Speciation.” Genetics 173, no. 1: 419–434. 10.1534/genetics.105.047985. 16204214 PMC1461441

[eva70268-bib-0084] Klopfenstein, D. V. , L. Zhang , B. S. Pedersen , et al. 2018. “GOATOOLS: A Python Library for Gene Ontology Analyses.” Scientific Reports 8, no. 1: 10872. 10.1038/s41598-018-28948-z. 30022098 PMC6052049

[eva70268-bib-0085] Korneliussen, T. S. , A. Albrechtsen , and R. Nielsen . 2014. “ANGSD: Analysis of Next Generation Sequencing Data.” BMC Bioinformatics 15: 356. 10.1186/s12859-014-0356-4. 25420514 PMC4248462

[eva70268-bib-0086] Laikre, L. , and N. Ryman . 1996. “Effects on Intraspecific Biodiversity From Harvesting and Enhancing Natural Populations.” Ambio 25, no. 8: 504–509. https://www.jstor.org/stable/4314530.

[eva70268-bib-0087] Lawrence, M. , W. Huber , H. Pagès , et al. 2013. “Software for Computing and Annotating Genomic Ranges.” PLoS Computational Biology 9, no. 8: e1003118. 10.1371/journal.pcbi.1003118. 23950696 PMC3738458

[eva70268-bib-0088] Lehnert, S. J. , I. R. Bradbury , B. F. Wringe , M. Van Wyngaarden , and P. Bentzen . 2023. “Multifaceted Framework for Defining Conservation Units: An Example From Atlantic Salmon ( *Salmo salar* ) in Canada.” Evolutionary Applications 16, no. 9: 1568–1585. 10.1111/eva.13587. 37752960 PMC10519414

[eva70268-bib-0089] Li, H. , and R. Durbin . 2009. “Fast and Accurate Short Read Alignment With Burrows–Wheeler Transform.” Bioinformatics 25, no. 14: 1754–1760. 10.1093/bioinformatics/btp324. 19451168 PMC2705234

[eva70268-bib-0090] Li, H. , and P. Ralph . 2019. “Local PCA Shows How the Effect of Population Structure Differs Along the Genome.” Genetics 211, no. 1: 289–304. 10.1534/genetics.118.301747. 30459280 PMC6325702

[eva70268-bib-0091] Li, Y.‐L. , and J.‐X. Liu . 2018. “StructureSelector: A Web‐Based Software to Select and Visualize the Optimal Number of Clusters Using Multiple Methods.” Molecular Ecology Resources 18, no. 1: 176–177. 10.1111/1755-0998.12719. 28921901

[eva70268-bib-0092] Linderoth, T. P. 2018. *Identifying Population Histories, Adaptive Genes, and Genetic Duplication From Population‐Scale Next Generation Sequencing* [Ph.D. Thesis, University of California, Berkeley]. https://escholarship.org/uc/item/5kp4q40k.

[eva70268-bib-0093] Liu, X. , N. M. Kopelman , and N. A. Rosenberg . 2024. “Clumppling: Cluster Matching and Permutation Program With Integer Linear Programming.” Bioinformatics 40, no. 1: btad751. 10.1093/bioinformatics/btad751. 38096585 PMC10766593

[eva70268-bib-0094] Lotterhos, K. E. 2019. “The Effect of Neutral Recombination Variation on Genome Scans for Selection.” G3: Genes, Genomes, Genetics 9, no. 6: 1851–1867. 10.1534/g3.119.400088. 30971391 PMC6553532

[eva70268-bib-0095] Lou, R. N. , A. Jacobs , A. P. Wilder , and N. O. Therkildsen . 2021. “A Beginner's Guide to Low‐Coverage Whole Genome Sequencing for Population Genomics.” Molecular Ecology 30, no. 23: 5966–5993. 10.1111/mec.16077. 34250668

[eva70268-bib-0096] Luck, G. W. , G. C. Daily , and P. R. Ehrlich . 2003. “Population Diversity and Ecosystem Services.” Trends in Ecology & Evolution 18, no. 7: 331–336. 10.1016/S0169-5347(03)00100-9.

[eva70268-bib-0097] MacKenzie, C. J. A. , B. L. Fortin , and C. E. Stevens . 2022. *Summary of Ecological Information Relevant to Great Slave Lake Fisheries* (Canadian Manuscript Report of Fisheries and Aquatic Sciences 3214; p. vii+63p). https://publications.gc.ca/collections/collection_2022/mpo‐dfo/Fs97‐4‐3214‐eng.pdf.

[eva70268-bib-0098] Mantel, N. 1967. “The Detection of Disease Clustering and a Generalized Regression Approach.” Cancer Research 27, no. 2: 209–220. 6018555

[eva70268-bib-0099] Matschiner, M. , J. M. I. Barth , O. K. Tørresen , et al. 2022. “Supergene Origin and Maintenance in Atlantic Cod.” Nature Ecology & Evolution 6, no. 4: 469–481. 10.1038/s41559-022-01661-x. 35177802 PMC8986531

[eva70268-bib-0100] McKenna, A. , M. Hanna , E. Banks , et al. 2010. “The Genome Analysis Toolkit: A MapReduce Framework for Analyzing Next‐Generation DNA Sequencing Data.” Genome Research 20, no. 9: 1297–1303. 10.1101/gr.107524.110. 20644199 PMC2928508

[eva70268-bib-0101] McKinney, G. J. , C. E. Pascal , W. D. Templin , et al. 2020. “Dense SNP Panels Resolve Closely Related Chinook Salmon Populations.” Canadian Journal of Fisheries and Aquatic Sciences 77, no. 3: 451–461. 10.1139/cjfas-2019-0067.

[eva70268-bib-0102] McLeod, C. , G. Ash , D. Fernet , et al. 1985. Fall Fish Spawning Habitat Survey, 1978–1985, 102. RL&L/EMA Slave River Joint Venture.

[eva70268-bib-0103] Mee, J. A. , L. Bernatchez , J. D. Reist , S. M. Rogers , and E. B. Taylor . 2015. “Identifying Designatable Units for Intraspecific Conservation Prioritization: A Hierarchical Approach Applied to the Lake Whitefish Species Complex (Coregonus spp.).” Evolutionary Applications 8, no. 5: 423–441. 10.1111/eva.12247. 26029257 PMC4430767

[eva70268-bib-0104] Meisner, J. , and A. Albrechtsen . 2018. “Inferring Population Structure and Admixture Proportions in Low‐Depth NGS Data.” Genetics 210, no. 2: 719–731. 10.1534/genetics.118.301336. 30131346 PMC6216594

[eva70268-bib-0105] Meisner, J. , A. Albrechtsen , and K. Hanghøj . 2021. “Detecting Selection in Low‐Coverage High‐Throughput Sequencing Data Using Principal Component Analysis.” BMC Bioinformatics 22, no. 1: 470. 10.1186/s12859-021-04375-2. 34587903 PMC8480091

[eva70268-bib-0106] Mérot, C. , E. L. Berdan , H. Cayuela , et al. 2021. “Locally Adaptive Inversions Modulate Genetic Variation at Different Geographic Scales in a Seaweed Fly.” Molecular Biology and Evolution 38, no. 9: 3953–3971. 10.1093/molbev/msab143. 33963409 PMC8382925

[eva70268-bib-0107] Mérot, C. , K. S. R. Stenløkk , C. Venney , et al. 2023. “Genome Assembly, Structural Variants, and Genetic Differentiation Between Lake Whitefish Young Species Pairs (Coregonus sp.) With Long and Short Reads.” Molecular Ecology 32, no. 6: 1458–1477. 10.1111/mec.16468. 35416336

[eva70268-bib-0108] Miller, M. , C. Stevens , and M. S. Poesch . 2025. “Effectiveness of Spawning Substrate Enhancement for Adfluvial Fish in a Regulated Sub‐Arctic River.” River Research and Applications 41, no. 2: 412–425. 10.1002/rra.4358.

[eva70268-bib-0109] Molton, K. J. , T. O. Brenden , and J. R. Bence . 2013. “Harvest Levels That Conserve Spawning Biomass Can Provide Larger and More Stable and Sustainable Yields in Intermixed Fisheries.” Fisheries Research 147: 264–283. 10.1016/j.fishres.2013.07.004.

[eva70268-bib-0110] Mouselimis, L. 2024. *ClusterR: Gaussian Mixture Models, K‐Means, Mini‐Batch‐Kmeans, K‐Medoids and Affinity Propagation Clustering* (Version R Package Version 1.3.3) [Computer Software]. https://cran.r‐project.org/web/packages/ClusterR/ClusterR.pdf.

[eva70268-bib-0111] Muir, A. M. , P. Vecsei , M. Power , C. C. Krueger , and J. D. Reist . 2014. “Morphology and Life History of the Great Slave Lake Ciscoes (Salmoniformes: Coregonidae).” Ecology of Freshwater Fish 23, no. 3: 453–469. 10.1111/eff.12098.

[eva70268-bib-0112] Nathan, L. , B. Sloss , J. Vandehey , et al. 2016. “Temporal Stability of Lake Whitefish Genetic Stocks in Lake Michigan.” Journal of Great Lakes Research 42, no. 2: 433–439. 10.1016/j.jglr.2016.01.006.

[eva70268-bib-0113] Nielsen, E. E. , J. Hemmer‐Hansen , P. F. Larsen , and D. Bekkevold . 2009. “Population Genomics of Marine Fishes: Identifying Adaptive Variation in Space and Time.” Molecular Ecology 18, no. 15: 3128–3150. 10.1111/j.1365-294X.2009.04272.x. 19627488

[eva70268-bib-0114] Nikolic, N. , F. Devloo‐Delva , D. Bailleul , et al. 2023. “Stepping up to Genome Scan Allows Stock Differentiation in the Worldwide Distributed Blue Shark *Prionace glauca* .” Molecular Ecology 32, no. 5: 1000–1019. 10.1111/mec.16822. 36511846

[eva70268-bib-0115] Northcote, T. G. 1997. “Potamodromy in Salmonidae—Living and Moving in the Fast Lane.” North American Journal of Fisheries Management 17, no. 4: 1029–1045. 10.1577/1548-8675(1997)017<;1029:PISAMI>;2.3.CO;2.

[eva70268-bib-0116] Nosil, P. , D. J. Funk , and D. Ortiz‐Barrientos . 2009. “Divergent Selection and Heterogeneous Genomic Divergence.” Molecular Ecology 18, no. 3: 375–402. 10.1111/j.1365-294X.2008.03946.x. 19143936

[eva70268-bib-0117] Nosil, P. , T. H. Vines , and D. J. Funk . 2005. “Reproductive Isolation Caused by Natural Selection Against Immigrants From Divergent Habitats.” Evolution 59, no. 4: 705–719. 10.1111/j.0014-3820.2005.tb01747.x. 15926683

[eva70268-bib-0118] Oksanen, J. , G. Simpson , F. Blanchet , et al. 2022. *vegan: Community Ecology Package* (Version 2.6‐4) [R]. https://CRAN.R‐project.org/package=vegan.

[eva70268-bib-0119] Pedersen, B. S. , and A. R. Quinlan . 2018. “Mosdepth: Quick Coverage Calculation for Genomes and Exomes.” Bioinformatics 34, no. 5: 867–868. 10.1093/bioinformatics/btx699. 29096012 PMC6030888

[eva70268-bib-0120] Potvin, C. , and L. Bernatchez . 2001. “Lacustrine Spatial Distribution of Landlocked Atlantic Salmon Populations Assessed Across Generations by Multilocus Individual Assignment and Mixed‐Stock Analyses.” Molecular Ecology 10, no. 10: 2375–2388. 10.1046/j.0962-1083.2001.01374.x. 11703650

[eva70268-bib-0121] Puechmaille, S. J. 2016. “The Program Structure Does Not Reliably Recover the Correct Population Structure When Sampling Is Uneven: Subsampling and New Estimators Alleviate the Problem.” Molecular Ecology Resources 16, no. 3: 608–627. 10.1111/1755-0998.12512. 26856252

[eva70268-bib-0122] Quilodrán, C. S. , K. Ruegg , A. T. Sendell‐Price , E. C. Anderson , T. Coulson , and S. M. Clegg . 2020. “The Multiple Population Genetic and Demographic Routes to Islands of Genomic Divergence.” Methods in Ecology and Evolution 11, no. 1: 6–21. 10.1111/2041-210X.13324.

[eva70268-bib-0123] Quinlan, A. R. , and I. M. Hall . 2010. “BEDTools: A Flexible Suite of Utilities for Comparing Genomic Features.” Bioinformatics 26, no. 6: 841–842. 10.1093/bioinformatics/btq033. 20110278 PMC2832824

[eva70268-bib-0124] Rasmussen, M. S. , G. Garcia‐Erill , T. S. Korneliussen , C. Wiuf , and A. Albrechtsen . 2022. “Estimation of Site Frequency Spectra From Low‐Coverage Sequencing Data Using Stochastic EM Reduces Overfitting, Runtime, and Memory Usage.” Genetics 222, no. 4: iyac148. 10.1093/genetics/iyac148. 36173322 PMC9713400

[eva70268-bib-0125] Rawson, D. S. 1947. “Chapter 5—Great Slave Lake.” In North West Canadian Fisheries Surveys in 1944‐1945, 45–68. https://waves‐vagues.dfo‐mpo.gc.ca/Library/35870.pdf.

[eva70268-bib-0126] Rawson, D. S. 1949. “Estimating the Fish Production of Great Slave Lake.” Transactions of the American Fisheries Society 77, no. 1: 81–92. 10.1577/1548-8659(1947)77[81:ETFPOG]2.0.CO;2.

[eva70268-bib-0127] Rawson, D. S. 1950. “The Physical Limnology of Great Slave Lake.” Journal of the Fisheries Research Board of Canada 8a, no. 1: 3–66. 10.1139/f50-001.

[eva70268-bib-0128] Rawson, D. S. 1951. “Studies of the Fish of Great Slave Lake.” Journal of the Fisheries Research Board of Canada 8b, no. 4: 207–240. 10.1139/f50-014.

[eva70268-bib-0129] Rawson, D. S. 1953. “The Bottom Fauna of Great Slave Lake.” Journal of the Fisheries Research Board of Canada 10, no. 8: 496–519. 10.1139/f53-028.

[eva70268-bib-0130] Rawson, D. S. 1956. “The Net Plankton of Great Slave Lake.” Journal of the Fisheries Research Board of Canada 13, no. 1: 53–127. 10.1139/f56-006.

[eva70268-bib-0131] Richardson, E. S. , J. D. Reist , and C. K. Minns . 2001. Life History Characteristics of Freshwater Fishes Occurring in the Northwest Territories and Nunavut, With Major Emphasis on Lake Habitat Requirements (Canadian Manuscript Report of Fisheries and Aquatic Sciences 2569). Fisheries and Oceans Canada. https://publications.gc.ca/collections/collection_2007/dfo‐mpo/Fs97‐4‐2569E.pdf.

[eva70268-bib-0132] Roberge, M. M. , G. Low , and C. J. Read . 1985. Investigation of a Fall Spawning Run of Lake Whitefish Into the Little Buffalo River Northwest Territories Canada. Canadian Manuscript Report of Fisheries and Aquatic Sciences, 1820. https://waves‐vagues.dfo‐mpo.gc.ca/Library/17900.pdf.

[eva70268-bib-0133] Rogers, S. M. 2008. Designatable Units at an Appropriate Scale for the Lake Whitefish, Coregonus clupeaformis in Canada (p. 64) [COSEWIC Special Report]. Comittee on the Status of Endangered Wildlife in Canada.

[eva70268-bib-0134] Rousset, F. 1997. “Genetic Differentiation and Estimation of Gene Flow From F‐Statistics Under Isolation by Distance.” Genetics 145, no. 4: 1219–1228. 10.1093/genetics/145.4.1219. 9093870 PMC1207888

[eva70268-bib-0135] Ryther, C. M. , R. Lauzon , M.‐C. Buell , R. Duncan , B. Redford , and E. S. Dunlop . 2024. “Spawning Behaviour of Lake Whitefish in Lake Huron Revealed by Fine‐Scale Acoustic Telemetry and Indigenous Ecological Knowledge.” International Journal of Limnology 60: 8. 10.1051/limn/2024007.

[eva70268-bib-0136] Samuk, K. , G. L. Owens , K. E. Delmore , S. E. Miller , D. J. Rennison , and D. Schluter . 2017. “Gene Flow and Selection Interact to Promote Adaptive Divergence in Regions of Low Recombination.” Molecular Ecology 26, no. 17: 4378–4390. 10.1111/mec.14226. 28667780

[eva70268-bib-0137] Schindler, D. E. , R. Hilborn , B. Chasco , et al. 2010. “Population Diversity and the Portfolio Effect in an Exploited Species.” Nature 465, no. 7298: 7298. 10.1038/nature09060. 20520713

[eva70268-bib-0138] Schneller, N. M. , J. M. Strugnell , M. A. Field , K. Johannesson , and I. Cooke . 2025. “Putting Structural Variants Into Practice: The Role of Chromosomal Inversions in the Management of Marine Environments.” Molecular Ecology 34: e17776. 10.1111/mec.17776. 40342214 PMC12684361

[eva70268-bib-0139] Schraidt, C. E. , A. S. Ackiss , W. A. Larson , M. D. Rowe , T. O. Höök , and M. R. Christie . 2023. “Dispersive Currents Explain Patterns of Population Connectivity in an Ecologically and Economically Important Fish.” Evolutionary Applications 16, no. 7: 1284–1301. 10.1111/eva.13567. 37492152 PMC10363847

[eva70268-bib-0140] Scott, D. C. , and R. R. Wheaton . 1954. A Study of Great Slave Lake at the Spawning Time of Lake Trout, Cristivomer Namaycush and Whitefish, Coregonus clupeaformis in 1953 With a Similar Study in 1952 as an Appendix (Manuscript Reports of the Biological Stations No. 565). Fisheries Research Board of Canada. https://waves‐vagues.dfo‐mpo.gc.ca/library‐bibliotheque/36883.pdf.

[eva70268-bib-0142] Shi, Y. , K. L. Bouska , G. J. McKinney , et al. 2023. “Gene Flow Influences the Genomic Architecture of Local Adaptation in Six Riverine Fish Species.” Molecular Ecology 32, no. 7: 1549–1566. 10.1111/mec.16317. 34878685

[eva70268-bib-0143] Shi, Y. , J. J. Homola , P. T. Euclide , et al. 2022. “High‐Density Genomic Data Reveal Fine‐Scale Population Structure and Pronounced Islands of Adaptive Divergence in Lake Whitefish ( *Coregonus clupeaformis* ) From Lake Michigan.” Evolutionary Applications 15, no. 11: 1776–1791. 10.1111/eva.13475. 36426119 PMC9679245

[eva70268-bib-0144] Skotte, L. , T. S. Korneliussen , and A. Albrechtsen . 2013. “Estimating Individual Admixture Proportions From Next Generation Sequencing Data.” Genetics 195, no. 3: 693–702. 10.1534/genetics.113.154138. 24026093 PMC3813857

[eva70268-bib-0145] Slingsby, C. , G. J. Wistow , and A. R. Clark . 2013. “Evolution of Crystallins for a Role in the Vertebrate Eye Lens.” Protein Science: A Publication of the Protein Society 22, no. 4: 367–380. 10.1002/pro.2229. 23389822 PMC3610043

[eva70268-bib-0146] Stenløkk, K. , M. Saitou , L. Rud‐Johansen , et al. 2022. “The Emergence of Supergenes From Inversions in Atlantic Salmon.” Philosophical Transactions of the Royal Society B: Biological Sciences 377, no. 1856: 20210195. 10.1098/rstb.2021.0195. PMC9189505 35694753

[eva70268-bib-0147] Stewart, D. B. 1999. “*A Review of Information on Fish Stocks And Harvests in the South Slave Area, Northwest Territories* (Canadian Manuscript Report of Fisheries and Aquatic Sciences 2493; p. 65).” https://publications.gc.ca/collections/collection_2007/dfo‐mpo/Fs97‐4‐2493E.pdf.

[eva70268-bib-0148] Storey, J. D. , A. J. Bass , A. Dabney , and D. Robinson . 2024. “qvalue: Q‐Value Estimation for False Discovery Rate Control. (Version 2.38.0) [Computer Software].” https://github.com/jdstorey/qvalue.

[eva70268-bib-0150] Tallman, R. , K. Howland , G. Low , W. Tonn , and A. Little . 2005. “Composition and Changes to the Fish Assemblage in a Large Sub‐Arctic Drainage: The Lower Slave River.” American Fisheries Society Symposium: 23–39. 10.47886/9781888569728.ch3.

[eva70268-bib-0151] Tallman, R. F. 1996. Synthesis of Fish Distribution, Movements, Critical Habitat and Food Web for the Lower Slave River North of the 60th Parallel: A Food Chain Perspective (Northern River Basins Study Synthesis Report 13; p. 170). Northern River Basins Study. https://publications.gc.ca/site/eng/61045/publication.html.

[eva70268-bib-0152] The UniProt Consortium . 2025. “UniProt: The Universal Protein Knowledgebase in 2025.” Nucleic Acids Research 53, no. D1: D609–D617. 10.1093/nar/gkae1010. 39552041 PMC11701636

[eva70268-bib-0153] Therkildsen, N. O. , and S. R. Palumbi . 2017. “Practical Low‐Coverage Genomewide Sequencing of Hundreds of Individually Barcoded Samples for Population and Evolutionary Genomics in Nonmodel Species.” Molecular Ecology Resources 17, no. 2: 194–208. 10.1111/1755-0998.12593. 27496322

[eva70268-bib-0154] Thorstensen, M. J. , J. D. Jeffrey , Y. Shi , et al. 2022. “A Chromosomal Inversion May Facilitate Adaptation Despite Periodic Gene Flow in a Freshwater Fish.” Ecology and Evolution 12, no. 5: e8898. 10.1002/ece3.8898. 35571758 PMC9077824

[eva70268-bib-0155] Tigano, A. , and V. L. Friesen . 2016. “Genomics of Local Adaptation With Gene Flow.” Molecular Ecology 25, no. 10: 2144–2164. 10.1111/mec.13606. 26946320

[eva70268-bib-0156] Tripp, D. B. , P. J. McCart , R. D. Saunders , and G. W. Hughes . 1981. Fisheries Studies in the Slave River Delta, NWT ‐ Final Report (Aquatic Environments Limited, p. 262). Mackenzie River Basin Study.

[eva70268-bib-0157] Turgeon, J. , and A. Bourret . 2013. “Genetic Differentiation and Origin of the Shortjaw Cisco ( *Coregonus zenithicus* ) in the Great Lakes and Other Inland Canadian Lakes.” (DFO Canadian Science Advisory Secretariat (CSAS) Research Document 2013/046; p. 37). https://publications.gc.ca/collections/collection_2013/mpo‐dfo/Fs70‐5‐2013‐046‐eng.pdf.

[eva70268-bib-0158] Turgeon, J. , S. M. Reid , A. Bourret , et al. 2016. “Morphological and Genetic Variation in Cisco ( *Coregonus artedi* ) and Shortjaw Cisco ( *C. zenithicus* ): Multiple Origins of Shortjaw Cisco in Inland Lakes Require a Lake‐Specific Conservation Approach.” Conservation Genetics 17, no. 1: 45–56. 10.1007/s10592-015-0759-4.

[eva70268-bib-0159] Utne‐Palm, A. C. 2002. “Visual Feeding of Fish in a Turbid Environment: Physical and Behavioural Aspects.” Marine and Freshwater Behaviour and Physiology 35, no. 1–2: 111–128. 10.1080/10236240290025644.

[eva70268-bib-0160] van Etten, J. 2017. “R Package Gdistance: Distances and Routes on Geographical Grids.” Journal of Statistical Software 76: 1–21. 10.18637/jss.v076.i13.36568334

[eva70268-bib-0161] Van Nynatten, A. , A. T. Duncan , R. Lauzon , et al. 2024. “Adaptive Evolution of Nearctic Deepwater Fish Vision: Implications for Assessing Functional Variation for Conservation.” Molecular Biology and Evolution 41, no. 2: msae024. 10.1093/molbev/msae024. 38314890 PMC10896662

[eva70268-bib-0162] Via, S. 2012. “Divergence Hitchhiking and the Spread of Genomic Isolation During Ecological Speciation‐With‐Gene‐Flow.” Philosophical Transactions of the Royal Society, B: Biological Sciences 367, no. 1587: 451–460. 10.1098/rstb.2011.0260. PMC3233719 22201174

[eva70268-bib-0163] Walker, S. H. , M. W. Prout , W. W. Taylor , and S. R. Winterstein . 1993. “Population Dynamics and Management of Lake Whitefish Stocks in Grand Traverse Bay, Lake Michigan.” North American Journal of Fisheries Management 13, no. 1: 73–85. 10.1577/1548-8675(1993)013<;0073:PDAMOL>;2.3.CO;2.

[eva70268-bib-0164] Wang, L. , Z. Y. Wan , H. S. Lim , and G. H. Yue . 2016. “Genetic Variability, Local Selection and Demographic History: Genomic Evidence of Evolving Towards Allopatric Speciation in Asian Seabass.” Molecular Ecology 25, no. 15: 3605–3621. 10.1111/mec.13714. 27262162

[eva70268-bib-0165] Waples, R. S. 1998. “Separating the Wheat From the Chaff: Patterns of Genetic Differentiation in High Gene Flow Species.” Journal of Heredity 89, no. 5: 438–450. 10.1093/jhered/89.5.438.

[eva70268-bib-0166] Waples, R. S. , K. A. Naish , and C. R. Primmer . 2020. “Conservation and Management of Salmon in the Age of Genomics.” Annual Review of Animal Biosciences 8, no. 1: 117–143. 10.1146/annurev-animal-021419-083617. 31730428

[eva70268-bib-0167] Weadick, C. J. , and B. S. W. Chang . 2009. “Molecular Evolution of the βγ Lens Crystallin Superfamily: Evidence for a Retained Ancestral Function in γN Crystallins?” Molecular Biology and Evolution 26, no. 5: 1127–1142. 10.1093/molbev/msp028. 19233964

[eva70268-bib-0168] Wellenreuther, M. , and L. Bernatchez . 2018. “Eco‐Evolutionary Genomics of Chromosomal Inversions.” Trends in Ecology & Evolution 33, no. 6: 427–440. 10.1016/j.tree.2018.04.002. 29731154

[eva70268-bib-0169] Wiens, L. N. , R. Bajno , J. T. Detwiler , M. Y. Janjua , and R. F. Tallman . 2021. “Genetic Assessment of Inconnu ( *Stenodus leucichthys* ) in Great Slave Lake, Northwest Territories, Canada.” Fisheries Research 234: 105784. 10.1016/j.fishres.2020.105784.

[eva70268-bib-0170] Wilson, C. C. , A. P. Liskauskas , and K. M. Wozney . 2016. “Pronounced Genetic Structure and Site Fidelity Among Native Muskellunge Populations in Lake Huron and Georgian Bay.” Transactions of the American Fisheries Society 145, no. 6: 1290–1302. 10.1080/00028487.2016.1209556.

[eva70268-bib-0171] Wright, S. 1943. “Isolation by Distance.” Genetics 28, no. 2: 114–138. 10.1093/genetics/28.2.114. 17247074 PMC1209196

[eva70268-bib-0172] Xuereb, A. , Q. Rougemont , X. Dallaire , et al. 2022. “Re‐Evaluating Coho Salmon ( *Oncorhynchus kisutch* ) Conservation Units in Canada Using Genomic Data.” Evolutionary Applications 15, no. 11: 1925–1944. 10.1111/eva.13489. 36426130 PMC9679250

[eva70268-bib-0173] Yeaman, S. 2013. “Genomic Rearrangements and the Evolution of Clusters of Locally Adaptive Loci.” Proceedings of the National Academy of Sciences 110, no. 19: E1743–E1751. 10.1073/pnas.1219381110. PMC3651494 23610436

[eva70268-bib-0174] Yeaman, S. , S. Aeschbacher , and R. Bürger . 2016. “The Evolution of Genomic Islands by Increased Establishment Probability of Linked Alleles.” Molecular Ecology 25, no. 11: 2542–2558. 10.1111/mec.13611. 27206531

[eva70268-bib-0175] Zanghi, C. , and C. C. Ioannou . 2025. “The Impact of Increasing Turbidity on the Predator–Prey Interactions of Freshwater Fishes.” Freshwater Biology 70, no. 1: e14354. 10.1111/fwb.14354.

[eva70268-bib-0176] Zhu, X. , A. Chapelsky , T. J. Carmichael , et al. 2017. Establishment of Ecological Baseline Metrics for Integrated Ecomonitoring and Assessment of Cumulative Impacts on Great Slave Lake Fisheries Ecosystems (Canadian Technical Report of Fisheries and Aquatic Sciences 3223; Issue 3223, p. ix + 58 pp.). Department of Fisheries and Oceans. https://epe.lac‐bac.gc.ca/100/201/301/weekly_acquisitions_list‐ef/2017/17‐42/publications.gc.ca/collections/collection_2017/mpo‐dfo/Fs97‐6‐3223‐eng.pdf.

[eva70268-bib-0177] Zimmerman, M. S. , C. C. Krueger , and R. L. Eshenroder . 2006. “Phenotypic Diversity of Lake Trout in Great Slave Lake: Differences in Morphology, Buoyancy, and Habitat Depth.” Transactions of the American Fisheries Society 135, no. 4: 1056–1067. 10.1577/T05-237.1.

