# Supplementary material for: Genome‐Wide Population Structure of Lake Whitefish (Coregonus clupeaformis) in a Subarctic Great Lake
Source: Evol Appl. 2026 May 28;19(6):e70268. doi: 10.1111/eva.70268 (PMC13240244; doi:10.1111/eva.70268)
Supplement: Supplementary file 1 — Figure S1: Mean individual depth of coverage per sample at each sampling location. Individuals from the Hay River genetic clusters are grouped separately. Figure S2.1: Admixture evaluation as correlation of residuals in evalAdmix for K2 to K10. Figure S2.2: Admixture evaluation as correlation of residuals in evalAdmix for K11 to K15. Figure S3.1: Individual admixture proportions from K = 2 to K = 8. Each graph is the average iteration of the most supported mode according to Clumppling, based on the aggregation of 50 runs for each K. Figure S3.2: Individual admixture proportions from K = 9 to K = 15. Each graph is the average iteration of the most supported mode according to Clumppling, based on the aggregation of 50 runs for each K. Figure S4: Scree‐plot of the % of variance explained for each of the first 20 PC axes based on a matrix of covariance computed using 181,603 SNPs. [file EVA-19-e70268-s001.docx]

**Supplementary Material & Methods**

**Identification and filtering of putatively duplicated individuals.**

The samples from this project were also sequenced using a Genotyping-in-Thousands by sequencing (GTseq) panel of 379 SNPs (Beemelmanns, Bouchard, Michaelides et al. 2025) specifically designed for Lake Whitefish for future mixed-stock analyses. Using the GTscore pipeline (McKinney et al. 2020), we genotyped all individuals and detected potentially duplicated samples using the *polyGenResults_dupTest* function. Using this panel, it was easier to assess if potential lab errors might have resulted in samples being inadvertently sequenced twice*.*  As we could not make sure that this potential duplication was not caused by pipetting errors during DNA extractions or by manipulation errors during field work, we removed the potentially duplicated individuals from both the original lcWGR (this study) and the GTseq (future study) datasets. Both individuals from pairs of samples that shared > 90% common genotypes were removed from the analysis, as we could not assess whether contamination was the source of the duplicated individuals.

**Supplementary Figure 1 :** Mean Individual Depth of Coverage per Sample at each Sampling Location. Individuals from the Hay River genetic clusters are grouped separately.

**Supplementary Figure 2.1:** Admixture evaluation as correlation of residuals in evalAdmix for K2 to K10.

**Supplementary Figure 2.2:** Admixture evaluation as correlation of residuals in evalAdmix for K11 to K15.

**Supplementary Figure 3.1**: Individual admixture proportions from K=2 to K=8. Each graph is the average iteration of the most supported mode according to *Clumppling*, based on the aggregation of 50 runs for each K.

**Supplementary Figure 3.2:** Individual admixture proportions from K=9 to K=15. Each graph is the average iteration of the most supported mode according to *Clumppling*, based on the aggregation of 50 runs for each K.

**Supplementary Figure 4 :** Scree-plot of the % of variance explained for each of the first 20 PC axes based on a matrix of covariance computed using 181,603 SNPs.
